# A protocol for development of a microsimulation model platform to evaluate the potential benefits, harms, and cost-effectiveness of risk-tailored melanoma screening

**DOI:** 10.1371/journal.pone.0339177

**Published:** 2025-12-26

**Authors:** Kirstie G. McLoughlin, Caroline G. Watts, Stephen Wade, Amelia K. Smit, H. Peter Soyer, Pablo Fernandez-Peñas, David C. Whiteman, Pascale Guitera, Gillian Reyes-Marcelino, Karen Canfell, Anne E. Cust, Michael Caruana

**Affiliations:** 1 The Daffodil Centre, The University of Sydney, and Cancer Council New South Wales, Sydney, New South Wales, Australia; 2 Melanoma Institute Australia, The University of Sydney, Sydney, New South Wales, Australia; 3 Frazer Institute, The University of Queensland, Dermatology Research Centre, Brisbane, Queensland, Australia; 4 Dermatology Department, Princess Alexandra Hospital, Brisbane, Queensland, Australia; 5 The University of Sydney, Faculty of Medicine and Health, Westmead Clinical School, New South Wales, Australia; 6 Department of Dermatology, Westmead Hospital, Westmead, New South Wales, Australia; 7 Departments of Population Health and Computational Biology, QIMR Berghofer Medical Research Institute, Queensland, Australia; 8 Faculty of Medicine, The University of Queensland, Queensland, Australia; 9 Faculty of Medicine and Health, The University of Sydney, Sydney, New South Wales, Australia; 10 Sydney Melanoma Diagnostic Centre, Royal Prince Alfred Hospital, New South Wales, Australia,; 11 Sydney School of Public Health, The University of Sydney, Sydney, New South Wales, Australia; Centers for Disease Control and Prevention, UNITED STATES OF AMERICA

## Abstract

In populations of European descent, melanoma is a high burden cancer in terms of incidence and healthcare costs, with early detection linked to better prognosis. There is no organised population screening program for melanoma in most countries, as more information is required about the potential benefits, harms and costs of population-based screening to develop policy. To assess the cost-effectiveness of a potential risk-tailored organised melanoma screening program in Australia, we have developed a protocol for a comprehensive microsimulation model (Policy1-Melanoma) that can evaluate multiple potential screening strategies. We outline the development of Policy1-Melanoma, a natural history model developed to be flexibly used to evaluate a range of scenarios related to melanoma screening, diagnosis, surveillance and management. We specify the types of data sources used for calibration and validation of Policy1-Melanoma, and the steps in this process.

## Introduction

Melanoma accounts for nearly 2% of global cancer burden [1], and its incidence globally has been steadily increasing [2,3]. Early diagnosis of melanoma is associated with high survival. This has driven interest in screening programs for melanoma, although the mortality benefit, cost-effectiveness, and potential harms from overdiagnosis (diagnosis of disease that would not have caused any symptoms or death) and overtreatment remains uncertain mainly due to a lack of randomised controlled trial data [4]. The U.S. Preventive Services Task Force has recently concluded that there is insufficient evidence to “assess the balance of benefits and harms of visual skin examination […] to screen for skin cancer” [4]. An organised approach to screening for melanoma in line with the World Health Organisation’s framework for population-based screening [5–7] has not been established at a national level in any country except for Germany [8].

Whilst survival from late-stage melanoma has improved with new adjuvant therapies [9], these treatments are very costly with later stage disease costing more than 60 times that of earlier stage disease in Australia [10]. Offsetting the growing costs of advanced cancer therapy could, in theory, improve the cost-effectiveness of screening approaches, as there are lower healthcare costs associated with early diagnosis. A better understanding of the benefits, harms and costs associated with structured approaches to the early detection of melanoma, including an organised national screening program, is needed to inform health policy. This is particularly true in Australia, where melanoma incidence remains the highest in the world [3] and represents 11% of new invasive cancer diagnoses [11].

Microsimulation models are computer programs that simulate individual lifetimes and individual-specific events to model the possible impacts of various interventions. Individual-based simulations have the virtue of capturing heterogenous aspects of population risk, behaviour, and health care services implementation, as well as sex and histologically based differentials in risk, natural history, and outcomes. This is particularly important in the context of melanoma screening as lifetime events (such as previous melanoma diagnosis or specific histological characteristics) can increase or decrease the chance of future events (like diagnosis or melanoma-specific death). Such models are particularly useful in assessing the utility of public health interventions [12,13]. Given the difficulty, time, and cost of conducting large-scale screening trials with long-term follow-up, detailed mathematical models that simulate cancer burden in the population are often used as a valuable source of quantification in policy decision making. For example, the U.S. Preventive Services Task Force has used models based on primary data from trials, contextualised in relation to local factors such as expected screening and management approaches and local burden of disease, to assess the utility of a range of approaches to cervical, breast, colorectal, and lung cancer screening [14–17]. Here we present a protocol for the development, calibration, and validation of a microsimulation platform to predict melanoma burden. It has been developed to be flexibly used to evaluate a range of scenarios related to melanoma screening, diagnosis, surveillance and management, including to assess the cost-effectiveness of a potential risk-tailored organised melanoma screening program in Australia.

## Methods and analysis

**Fig 1:**
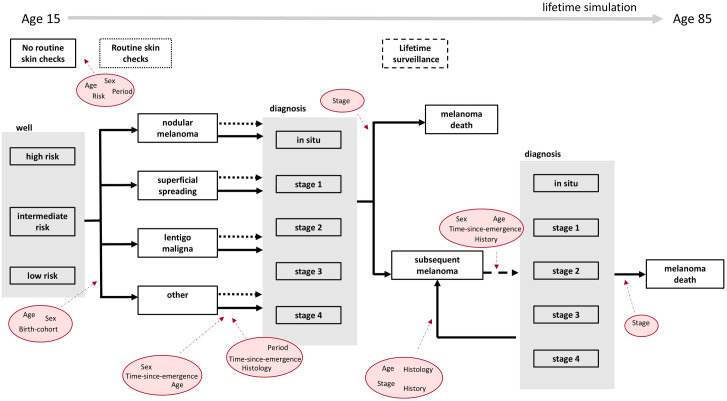
Schematic of the Policy1-Melanoma model structure. Individuals enter the simulation at age 15 in a healthy (‘well’) state having previously undertaken no routine skin checks. Over the course of their lifetime, they may develop a first primary melanoma with one of three specific histological subtypes or no specified histological subtype. At any point before diagnosis an individual can take up routine skin checks, which changes the probability of an undiagnosed melanoma being identified (dotted line). Melanoma diagnosis rates depend upon the sex, age, and skin check behaviour of the individual, their tumour histological subtype, and the period in which the identifying interaction (clinician visit) occurs. Tumours are classified using ICD-O-3 for body sites C44.0-C44.9 (and C80.9 melanoma of unknown primary site if not coded C44.9) and morphology codes M872-M879 with behaviour code 2 (in situ) or 3 (invasive). Once a first primary melanoma is diagnosed, a stage is allocated depending on tumour histological subtype and the time between its development and diagnosis. The individual may die of their melanoma, where survival is stage dependent. Most individuals do not die of their first melanoma and therefore have the potential to develop a subsequent primary melanoma. The probability of subsequent melanoma diagnosis is based on an assumption of lifetime surveillance.

Policy1-Melanoma is a new microsimulation platform in development from 2024–2026, using data inputs accessed from different sources including published data (see Data Inputs section and Table 1). Data from 1982 onwards were also obtained from the cancer registries for each state and territory in Australia, with ethics approval from the NSW Population and Health Service Research Ethics Committee (2020/ETH03109; data accessed from 01/01/2022). Through regular meetings with a multidisciplinary team, we developed a natural history structure of melanoma that captures unique aspects of this disease such as its capacity to be detected on the skin from visible inspection, and the risk of developing multiple primary melanomas (Fig 1) and allows us to disentangle the effects of screening and surveillance. This natural history structure addresses key limitations identified in a recent systematic review, which synthesised the structures, parameterisation, and assumptions of existing models [18]. This discrete event microsimulation involves both pre- and post-diagnosis lifetime events (natural history and treatment pathways, respectively) developed to be flexibly used in cost-effectiveness analyses of screening and surveillance in the management of melanoma. Policy1-Melanoma is implemented in C++ with an interface accessible using R. Reporting is guided by the checklist for health economic analysis plans based on Thorn et al (S1 Table) [19].

**Table 1:** Assumptions and justifications of Policy1-Melanoma for calibration and/or future evaluations of cutaneous melanoma diagnosis and management in Australia.

Transition	Assumptions	Justification and References
No skin checks to skin checks	In the context of population screening, screening usually refers to detection of a first primary cancer, thus individuals can only take up screening skin checks before their first primary melanoma is diagnosed.	After diagnosis of melanoma, skin checks form part of ‘surveillance’, not ‘screening’. However, both screening and surveillance can be modelled.
	Uptake of skin checks depends on age, sex, individual risk level, and calendar year.	The proportion of the Australian population screening has increased over time [32].
	Once skin checks are started, compliance may decline over time if there is no melanoma diagnosis.	Limited data available; we will estimate this through expert opinion and cohort studies, and/or through structured expert elicitation.
Risk groups	Lifetime melanoma risk is set at the start of the simulation. Individuals remain in the same relative risk group throughout their lifetime or until first melanoma diagnosis. Risk of melanoma development increases after an initial melanoma diagnosis.	Changes to potentially varying risk factors, e.g., family history, non-melanoma skin cancer diagnosis, mole count, are not incorporated into the model. The impact of age is incorporated within the ‘well’ to ‘undiagnosed first melanoma’ transition.
	Individual risk level changes the probability of melanoma emerging, and associations of individual risk with melanoma incidence remain constant over time.	[33]
	Individual risk level changes the uptake of skin checks and adherence over time.	Individuals at higher risk are more likely to attend screening programs [34] and opportunistic skin checks [32].
Well to undiagnosed first melanoma	First melanoma development depends on birth cohort, sex, individual risk level and age.	[33,35,36]
	Birth cohorts before 1972 have highest rates of melanoma development, between 1972 and 1992 rates decrease until the impact of sun protection campaigns is reached.	Sun protection behaviour campaigns in Australia started in 1982, particularly targeting children [27,29,37]. UV exposure influences melanoma risk more during childhood than adulthood [38,39].
	There is no difference in the probability of a specific histological subtype diagnosis between the sexes.	Unpublished data from Australian cancer registries.
	Relative proportion of histological subtypes has not changed over time, accounting for age.	Limited Australian data available (Australian Cancer Registries earliest state date in 1972).
	Individuals with any of the undiagnosed melanoma histologies have detectable melanoma. Before melanoma is detectable individuals remain “well”.	
Undiagnosed first melanoma to diagnosis.	Attending a clinical skin check varies with patients’ sex, age, individual risk level and time since melanoma development. Detection by a clinician depends on the melanoma histological subtype, calendar period of diagnosis, time since melanoma development, and the body site of the melanoma. Diagnosis depends on attending a clinical skin check and the ability of a clinician to detect a melanoma.	[32,40,41]
	Skin checks impact detection rates.	[42]
	Time left undiagnosed influences the stage at diagnosis: i.e., the longer the melanoma is undiagnosed the later the stage at diagnosis.	[21,43,44]
	The speed and direction of melanoma growth depends on histological subtype.	[21]
Undiagnosed first melanoma to melanoma-specific death.	Breslow thickness and stage at diagnosis affect melanoma-specific survival. Within stage I and II, tumour thickness influences survival. Prognosis in later stages (III and IV) is more related to the presence of regional and distant metastases.	[9,45]
Lifetime surveillance.	Surveillance depends on stage at diagnosis and risk of developing a new primary melanoma, with more frequent surveillance for those at higher stage and higher risk.	[46]
Diagnosed melanoma (either first or subsequent) to undiagnosed subsequent melanoma.	The chance of subsequent primary diagnosis depends on individual risk level, histological subtype of first primary melanoma, and number of previous melanomas. Time to subsequent diagnosis of new primary melanoma decreases with number of previous primary melanomas.	[47,48]
	Subsequent primaries are more likely than not to be the same histological subtype as the first primary melanoma.	[49,50]
Undiagnosed subsequent primary melanoma to diagnosis.	Surveillance skin checks after a first diagnosis is a six-monthly or annual event.	[46,51]
	At a surveillance skin check the probability of a diagnosis depends upon the time since development and histological subtype of the tumour.	[40]
	For diagnosis of a subsequent primary melanoma, the same probability of attending a clinician and detection applies as for a first melanoma.	Limited data available.
Diagnosed subsequent melanoma to melanoma-specific death.	Melanoma-specific death can occur from any previous invasive melanoma (not necessarily the most recent).	[52]
	Stage at diagnosis and Breslow thickness influence survival.	[53, 9, 45]

### Natural history structure

We model the development of first invasive or in situ melanomas by the three main melanoma histologies (superficial spreading, lentigo maligna, nodular) alongside an ‘other’ histology category that includes both unspecified and rarer subtypes, their diagnosis, and the potential for subsequent diagnoses of primary melanomas. These histopathological subtypes are incorporated into Policy1-Melanoma as they are associated with both the capacity for tumour detection and its growth and staging at diagnosis, and thus play an important role in understanding the potential utility of screening. For example, unlike superficial spreading and lentigo maligna melanomas that initially develop horizontally across the surface of the skin, nodular melanoma develops orthogonally to the skin and into the epidermis; this internally-directed growth means that nodular tumours are more difficult to visually detect at an early stage and are thus associated with a thicker Breslow thickness and later stage at diagnosis [20]. The progression through staging also varies between melanoma histologies; whilst a nodular melanoma might progress from in situ to stage 4 within a six-month period [21], a lentigo maligna melanoma might make this progress over the course of 20 years [22]. The corollary of this differential growth is that screening is likely to detect melanomas that already have a better prognosis, a phenomenon known as length-time bias [23].

Within Policy1-Melanoma, all individuals enter the simulation at age 15, in a ‘well’ state, with an associated lifetime risk of melanoma development and with no current skin check behaviour (Fig 1). The model uses a lifetime time horizon; individuals can develop various melanoma states (both natural history and post-diagnosis) until they reach 85 or die, from melanoma-specific or other causes. Competing events within Policy1-Melanoma will be dealt with using event-specific distributions; the time at which each competing event occurs is sampled from these distributions (calculated from transition probabilities listed in Table 1) and the earliest occurring event is selected [24]. The assumptions underlying each transition within Policy1-Melanoma are summarised in Table 1.

Another characteristic related to melanoma incidence, which must be incorporated into the mathematical model, is the change in sun protection behaviours over time [25–27]. Reducing UV exposure, especially in childhood, is associated with lower risk of single and multiple primary melanoma [28]. Since 1980, Australia has implemented many public health programs to reduce sun exposure, which have been credited with reducing melanoma incidence in people under the age of 40 years [29,30]. Some of the reduction in incidence may also be due to changes in the ancestral composition of the Australian population [31]. Nevertheless, despite the risk of being diagnosed with melanoma by the age of 30 in Australia halving since 1982, it remains the most common cancer among young adults aged 20–39 years and represents 16% of new invasive cancer diagnoses for that age-group [11]. To simulate the burden of melanoma within Australia, birth-cohort variations will be considered, using cancer registry and other published data. These preventative campaigns have also raised awareness of the early detection of melanoma and led to more prevalent opportunistic skin checks within the Australian population [32], which act as non-standardised screening events. When assessing the utility of a melanoma screening program, the comparison group should be considered carefully given that opportunistic screening may be widespread [32].

Melanoma development within Policy1-Melanoma therefore depends upon age, sex, and birth cohort parameters. Combined, these factors provide a transition rate into one of four histologies, mentioned above. The effect of individual risk is to scale the ‘well’ to ‘undiagnosed melanoma’ transition rate. Specifically, all individuals have a probability of developing a melanoma, which is selected from a distribution which is affected by age, sex, and birth cohort. The effect of these factors is assumed to be constant between the risk groups, but the starting transition probability varies such that individuals in the high-risk group are more likely to develop melanomas at any time point. Whilst undiagnosed, the tumour can continue to develop. There are no distinct states for undiagnosed tumours, instead progression is implicitly modelled by assuming that the longer an individual remains undiagnosed the more likely they are to be diagnosed at a later stage and with worse survival-associated characteristics. Diagnosis is thus dependent upon time-since-melanoma-emergence (Fig 1), and the histological characteristics of the tumour.

### Factors influencing detection of melanoma

There are unique factors associated with melanoma that are different to many other cancers, particularly that it is a cancer appearing on a visible surface (the skin), which impact the way we simulate melanoma development and diagnosis. The visibility of melanomas means that clinicians can, in theory, detect a melanoma incidentally with relative ease simply through seeing a patient during their normal practice [40]. The attendance of individuals to a doctor (such as a general practitioner) can, therefore, be expected to influence their rate of melanoma detection. For example, the slightly higher melanoma incidence in young women [54] may be partly an artefact of more frequent clinician interactions resulting in more detections in this age group rather than reflecting fewer extant cases in young men.

First melanoma diagnosis is thus affected by factors that influence visiting a clinician (for example private health insurance, socioeconomic status, age and sex) and also by factors that change the probability of detection once an individual is seen by a clinician; such as changes in detection technology over time, and the difference in difficulty diagnosing the specified melanoma histologies. Specifically, we note that whilst the likelihood of diagnosis of superficial spreading melanoma and lentigo maligna melanoma increase as they develop, there is not such an association for nodular melanomas due to their early and rapid vertical growth phase.

Diagnostic technologies have also improved, and usage has changed over time [55,56]. Dermoscopy has been shown to improve diagnostic sensitivity and reduce benign excisions among dermatologists and trained general practitioners [57–60]. Other technologies for diagnosing melanoma include total body photography incorporating 3D images, digital monitoring, in vivo reflectance confocal microscopy, and the use of machine learning algorithms [61]. The effect of changing diagnostic techniques is incorporated into Policy1-Melanoma as a scaling function, improving diagnostic rates until they match observed diagnostic technology usage in Australia. Screening within Policy1-Melanoma is implemented as a detection event. Individuals ‘attend’ a screening event at which, if they have an undiagnosed melanoma, melanoma can be diagnosed. The probability of melanoma being diagnosed at a screening event depends upon several factors including the stage, histology, and location of the undiagnosed tumour (location is assigned using appropriate probability distributions derived from relevant epidemiological data), as well as the sensitivity and specificity of any diagnostic procedures involved. The improved diagnostic rates from dermoscopy (or other diagnostic technologies) are also included as period effects in screening detection probability.

Once diagnosed, melanoma stage is defined depending upon the time-since emergence and tumour histology. There is no explicit stage progression after diagnosis – the potential for melanoma spread is incorporated implicitly into melanoma survival, alongside age, stage, and histological characteristics. Individuals who develop a first primary melanoma may develop a subsequent melanoma, die from advanced melanoma, or die from other causes (Fig 1).

### Incorporation of personal risk and multiple primary melanomas

Another unique aspect of melanoma is that an individual can develop multiple primary melanomas over their lifetime, with risk of a new primary melanoma increasing with each new diagnosis [48], although the number of melanomas developed does not necessarily worsen survival [53]. In Australia, the proportion of melanoma patients who develop a second primary melanoma over 10 years is about 16% [62], although the risk varies according to the presence of other risk factors such as genetic predisposition [48,63]. After an initial invasive melanoma diagnosis patients usually remain under heightened lifetime skin surveillance, with annual or more frequent skin checks although guidelines do not usually make a distinction between invasive or in situ melanoma for skin check surveillance recommendations [46,64]. Australian guidelines for follow-up for risk of recurrence are based on stage at diagnosis, with more intensive follow-up for patients with greater risk of recurrence and the frequency of follow-up visits decreasing over time [46,64]. Long-term surveillance after melanoma diagnosis can inflate the costs associated with a screening program without providing additional benefits. Within Policy1-Melanoma, multiple primary melanomas are therefore incorporated separately (see Fig 1). New primary melanomas have distinct stage and histology characteristics and occur sequentially. Over an individual’s lifetime there is no explicit limit on the number of multiple primary melanomas.

The development of a subsequent primary melanoma, in Policy1-Melanoma, depends upon the histological subtype of the first primary melanoma’s time since first diagnosed, as well as sex and personal risk level of the individual. The diagnosis of a subsequent primary melanoma is implemented as a surveillance event with diagnosis dependent upon tumour histology and patient history (including age, number of previous primary diagnoses, and personal risk). We allow compliance to surveillance to decay based on time-since-diagnosis, histology, and stage at first diagnosis. We assume there is no difference in disease progression rates between a first and subsequent primary melanoma. Instead, progression rates and associated risk of development depend upon histological subtype for both first and subsequent melanomas. Implementing lifetime surveillance after a first melanoma diagnosis enables the separation of the effects of different surveillance methods and costs from that of the initial screening event.

### Data inputs

There are many data inputs required for the microsimulation. A key input is age- and sex-specific melanoma incidence and mortality rates for first and subsequent primary melanomas from each of the eight Australian state and territory cancer registries, from 1982 to the latest available. Older birth cohorts will be used for calibrating and validating Policy1-Melanoma with historical data, and for conducting multi-cohort analyses. Melanoma is a notifiable disease in Australia, and reporting is required for each jurisdiction. Primary care datasets [65,66] will be used to inform the patterns of which the public visits primary care clinicians. Associated costs will be obtained from the Medicare Benefits Schedule (Australia’s national health insurance scheme including a list of health professional services that the Australian Government subsidises [67]). Population-based studies including the Melanoma Patterns of Care Study [40] and National Sun Protection Survey [32] will be used to inform the patterns in skin check behaviours over time. Incidence rates of subsequent primary melanoma will be identified through an analysis of the cancer registry data and other population-based studies. Mortality rates will be incorporated into Policy1-Melanoma using relative survival, estimated from the cancer registries, and fitted against the reported mortality rates in Australia. The QSkin cohort study is a large skin cancer cohort in Queensland, Australia, that will be used for validation of the model [68]. Systematic reviews will be identified where relevant for other data inputs such as melanoma risk prediction tools [69] and health utilities [70,71]. Melanoma risk prediction tools will be used to stratify personal risk levels and incorporated into Policy1-Melanoma to evaluate the potential impact of an organised risk-stratified melanoma screening program.

Other transition probability distributions will be informed through literature reviews or clinician elicitation. Not all transitions within Policy1-Melanoma have observable parameters, moreover some transitions have multiple factors contributing to their distribution (such as birth-cohort and period effects on detection rates). We will calibrate Policy1-Melanoma using a Bayesian synthesis approach [72,73]. This methodology follows a systematic process to integrate multiple data sources to estimate model parameters while quantifying associated uncertainties. We will first establish prior distributions for model parameters informed by existing literature and clinician consultation. Subsequently, likelihood functions will be employed to align simulated outputs with observed melanoma data, facilitating parameter updates via Bayesian inference. This approach enables the synthesis of heterogeneous data and yields uncertainty estimates for model outputs, ensuring a rigorous calibration process. Model refinement will involve iterative diagnostic evaluations and sensitivity analyses [72,73] to produce a well-calibrated model that accurately represents melanoma epidemiology. The uncertainty associated with parameter selection will be assessed through probabilistic sensitivity analyses and value of information analyses.

Keratinocyte cancers and benign lesions will not be explicitly simulated in the initial iteration of the model. However, when evaluating the cost-effectiveness of risk-based melanoma screening programs, we will consider the financial implications of diagnosing these cancers and lesions in individuals participating in the program. Specifically, for each simulated skin check, we will incorporate the associated costs of keratinocyte cancers and benign lesions based on their expected prevalence and treatment expenses [74]. Keratinocyte cancers and benign lesions will be incorporated into subsequent versions of the model.

### Costs of melanoma

A cost-effectiveness analysis will be conducted using a health system (payer) perspective and lifetime horizon [75,76]. Outcome measures will include the incremental cost effectiveness ratio per life years saved and the incremental cost effectiveness ratio per quality-adjusted life year gained, with quality of life weights sourced from existing literature [77,78]. The comparator for cost-effectiveness analysis would be the current situation in Australia (opportunistic screening).

Clinical pathways related to screening and management of skin cancer will be carefully mapped in consultation with skin cancer experts. Costs will be estimated from the year of screening and diagnosis, which would be assumed to occur within the same year. To source costs related to screening and management, the Medicare Benefits Schedule will be used. Where the costs of screening strategies include using technology currently not listed on the Schedule, such as for example digital dermoscopy imaging, the costs will be sourced from the published literature. For hospital management, the National Hospital Cost Data Collection Public Sector Report, which is published annually, will be used. Costs for the skin cancer screening program development and operation will be estimated from other Australian screening programs.

Costs and benefits will be discounted at 5%, and a willingness to pay threshold of AUD 50,000 per quality-adjusted life year will be applied as these values are typically used in submissions for health technology assessment reimbursement in Australia. Sensitivity analysis will be conducted to examine the assumptions in the model.

Based on the scenarios considered appropriate for appraisal by policy makers, a budget impact analysis to estimate the annual health system costs of a screening program over five years will also be conducted.

## Discussion

There are multiple applications to which Policy1-Melanoma can provide insights, including simulating risk-tailored screening, treatment, and surveillance pathways, quantifying the expected risk of overdiagnosis from screening and surveillance, and providing estimates on cost-effectiveness and resource implications of different pathways and on the impact of changing diagnostic and treatment options.

Given the inherent heterogeneity of melanoma risk, developing a model to assess the efficacy of a screening program necessitates a simulation that includes inter-person variability. Policy1-Melanoma conducts individual-based simulations, yet represents a simplification of the complex dynamics of melanoma growth in the Australian population. For example, many of the data inputs that contribute to the model development are overrepresented in people of European descent despite the increasingly ethnically diverse Australian population. Future iterations of screening models for Australia will need to incorporate the increasing heterogeneity of the Australian population, which will reply on collecting more data across population subgroups. While the proposed protocol is tailored to the Australian context, its structure can be adapted for other countries by validating the transition assumptions in Table 1 and parameterising the model with country-specific data.

Policy1-Melanoma will be used to provide evidence addressing the Australian Population Based Screening Framework [7] and Australian Cancer Plan [79] so that the Australian Government, and ultimately other countries, can assess the merits of a potential organised, risk-stratified, melanoma national screening program, which would sit alongside already established screening programs for breast, bowel, and cervical cancers.

## Supporting information

S1 Table Expected content of health economic analysis plans, based on Thorn et al. (DOCX)
