# Supplementary material for: A protocol for development of a microsimulation model platform to evaluate the potential benefits, harms, and cost-effectiveness of risk-tailored melanoma screening
Source: PLoS One. 2025 Dec 26;20(12):e0339177. doi: 10.1371/journal.pone.0339177 (PMC12742747; doi:10.1371/journal.pone.0339177)
Supplement: S1 Table — (DOCX) [file pone.0339177.s001.docx]

**Supplementary Table 1.** Expected content of health economic analysis plans, based on Thorn et al.

| **Item** | **Item status after final voting** | **Item included in protocol?** |
| --- | --- | --- |
| Title | IN | Yes. |
| Trial registration number | IN | NA – not a trial |
| Source of funding | IN | Yes - added |
| Purpose of health economics analysis plan (HEAP) | IN | NA –not a trial, but the Aims of the protocol are specified in the Abstract and Introduction |
| Sponsor approval | OUT | No |
| Trial protocol version | IN | NA – not a trial |
| Trial statistical analysis plan (SAP) version | IN | NA – not a trial |
| Trial HEAP version | IN | NA – not a trial |
| HEAP revisions | IN | NA – not a trial |
| Table of contents | OPTIONAL LIST | No |
| Abbreviations/glossary of terms/definitions | OPTIONAL LIST | No |
| Roles and responsibilities | IN | Yes. See Authors Contributions section. |
| Signature(s) of person(s) writing HEAP (and date) | IN | NA – not a trial |
| Signature of senior health economist (HE) who is guarantor of the economic evaluation (and date) | IN | NA – not a trial |
| Signature of the chief investigator for the trial | IN | NA – not a trial |
| Trial background and rationale | IN | NA. Background information in Introduction. |
| Aim(s) of the trial | IN | NA – this is not a trial, but the Aims of the protocol are specified in the Abstract and Introduction |
| Objectives and/or research hypotheses of the trial | IN | NA as above |
| Trial population | IN | Yes. See Introduction & Methods and analysis. |
| Intervention and comparator(s) | IN | NA. Protocol is for a simulation which can be used in multiple contexts. |
| Trial design | IN | NA – not a trial |
| Trial start and end dates | IN | NA – not a trial |
| Aim(s) of economic evaluation | IN | This is a protocol for a generalisable microsimulation model of melanoma that can be flexibly used to evaluate a range of scenarios related to melanoma screening, diagnosis, surveillance and management (rather than a specific economic evaluation). However, in the Abstract and Introduction we mention our specific aim to evaluate skin cancer screening. |
| Objectives(s)/hypotheses of economic evaluation | IN | As above |
| Overview of economic analysis | IN | Methods and Analysis |
| Jurisdiction | IN | Yes. See Introduction & Methods and analysis. |
| Perspective(s) | IN | Yes – see Costs of melanoma section. |
| Time horizon | IN | Yes – added lifetime time horizon. See Methods and analysis section – Natural history structure. |
| Monitoring collection of health economic data | OPTIONAL LIST | No. |
| Database management | OPTIONAL LIST | NA. |
| Data entry | OPTIONAL LIST | NA |
| Data cleaning for analysis | IN | No. Data was cleaned by the Australian Cancer Registries. Additional cleaning may be performed before economic analyses are performed and this information would be provided in the context of a published model and specific evaluations. |
| Data archiving | OPTIONAL LIST | NA |
| Statistical software used for HE analysis | IN | Yes - added. See Methods and analysis section. |
| Identification of resources | IN | Yes – see Costs of melanoma section. |
| Measurement of resource use data | IN | Yes - as above |
| Valuation of resource use data | IN | Yes - see Costs of melanoma section. |
| Identification of outcome(s) | IN | Yes - see Costs of melanoma section. |
| Measurement of outcome(s) | IN | Yes - see Costs of melanoma section. More details will be provided in later manuscripts showing results of specific evaluations. |
| Valuation of outcome(s) | IN | Yes - see Costs of melanoma section. More details will be provided in later manuscripts showing results of specific evaluations. |
| Analysis population | IN | See Methods and Analysis for broad population details |
| Timing of analyses | IN | No – as the analyses will be ongoing |
| Discount rates for costs and benefits | IN | Yes - see Costs of melanoma section. |
| Cost-effectiveness threshold(s) | IN | Yes - see Costs of melanoma section. |
| Statistical decision rule(s) | IN | NA – not a trial, will depend on specific evaluation |
| Analysis of resource use | IN | NA – not a trial, will depend on specific evaluation |
| Analysis of costs | IN | NA – not a trial, will depend on specific evaluation |
| Analysis of outcomes | IN | NA – not a trial, will depend on specific evaluation |
| Missing data | IN | NA – not a trial, will depend on specific evaluation |
| Analysis of cost-effectiveness | IN | Yes – broad details shown in Costs of melanoma section. |
| Sampling uncertainty | IN | NA – not a trial, will depend on specific evaluation |
| Subgroup analysis/Analysis of heterogeneity | IN | NA – not a trial, will depend on specific evaluation |
| Sensitivity analyses | IN | NA – not a trial, will depend on specific evaluation |
| Post hoc analyses | OUT | No. This manuscript is a protocol for model development and does not include specific cost-effectiveness or trial analyses. |
| Extrapolation or decision analytic modeling | IN | Yes. See Methods and analysis section. |
| Model type | IN | Yes. See Methods and analysis section. |
| Model structure | IN | Yes. See Figure 1. |
| Treatment effect beyond the end of the trial | IN | NA |
| Other key assumptions | IN | Yes. See Table 1. |
| Methods for identifying and estimating parameters | IN | Yes. See Methods and analysis (Data inputs) section. |
| Model uncertainty | IN | Yes. See Methods and analysis (Data inputs) section. |
| Model validation | IN | Yes. See Methods and analysis (Data inputs) section. |
| Subgroup analyses/Heterogeneity | IN | NA –will depend on specific evaluation |
| Value of information analysis | OPTIONAL LIST | No |
| Responsibility for health economic results and reporting | OUT | No |
| Reporting standards | IN | No. There are no results reported in this protocol paper. |
| Reporting deviations from the HEAP | IN | NA – not a trial |
| References to trial and statistical master file | OUT | NA – not a trial |
| References to other trial documents | OPTIONAL LIST | NA – not a trial |
| Appendices: Resource use data collected | IN | Table 1 and Costs of melanoma section |
| Appendices: Reporting checklists | OUT | No |
| Appendices: Illustrations | OPTIONAL LIST | Yes |

*Thorn JC, Davies CF, Brookes ST, Noble SM, Dritsaki M, Gray E, Hughes DA, Mihaylova B, Petrou S, Ridyard C, Sach T. Content of Health Economics Analysis Plans (HEAPs) for trial-based economic evaluations: expert Delphi consensus survey. Value in Health. 2021 Apr 1;24(4):539-47.).*
