# Supplementary material for: A protocol for development of a microsimulation model platform to evaluate the potential benefits, harms, and cost-effectiveness of risk-tailored melanoma screening
Source: PLoS One. 2025 Dec 26;20(12):e0339177. doi: 10.1371/journal.pone.0339177 (PMC12742747; doi:10.1371/journal.pone.0339177)

PLOS ONE

A protocol for development of a microsimulation model platform to evaluate the potential benefits, harms, and cost-effectiveness of risk-tailored melanoma screening

--Manuscript Draft--

**Manuscript Number:**	PONE-D-24-40002
**Article Type:**	Study Protocol
**Full Title:**	A protocol for development of a microsimulation model platform to evaluate the potential benefits, harms, and cost-effectiveness of risk-tailored melanoma screening
**Short Title:**	A protocol for development of a microsimulation model platform to evaluate risk-tailored melanoma screening
**Corresponding Author:**	Anne Elizabeth Cust, PhD, MPH(Hons), BSc, BA<br>University of Sydney<br>Newtown, NSW AUSTRALIA
**Keywords:**	melanoma; microsimulation; screening; surveillance; skin check; economic model; Risk Assessment; health decision modelling; skin cancer
**Abstract:**	In populations of European descent, melanoma is a high burden cancer in terms of incidence and healthcare costs, with early detection linked to better prognosis. There is no organised population screening program for melanoma in most countries, as more information is required about the potential benefits, harms and costs of population-based screening to develop policy. To assess the cost-effectiveness of potential risk-tailored organised melanoma screening programs, we have developed a protocol for a comprehensive microsimulation model (Policy1-Melanoma) that can evaluate multiple potential screening strategies. We outline the development of Policy1-Melanoma, a natural history model that will evaluate whether a risk-tailored organised melanoma screening program would be cost-effective in Australia. We specify the types of data sources used for calibration and validation of Policy1-Melanoma, and the steps in this process.
**Order of Authors:**	Kirstie G McLoughlin
	Caroline G Watts
	Stephen Wade
	Amelia K Smit
	H. Peter Soyer
	Pablo Fernandez-Peñas
	David C. Whiteman
	Pascale Guitera
	Gillian Reyes-Marcelino
	Karen Canfell
	Anne Elizabeth Cust, PhD, MPH(Hons), BSc, BA
	Michael Caruana
**Additional Information:**	
**Question**	**Response**
**Financial Disclosure**<br><br>Enter a financial disclosure statement that describes the sources of funding for the work included in this submission. Review the submission guidelines for detailed	Yes

Powered by Editorial Manager® and ProduXion Manager® from Aries Systems Corporation

requirements. View published research articles from *PLOS ONE* for specific examples.<br><br>This statement is required for submission and **will appear in the published article** if the submission is accepted. Please make sure it is accurate.<br><br>**Funded studies**<br>Enter a statement with the following details:<br>• Initials of the authors who received each award<br>• Grant numbers awarded to each author<br>• The full name of each funder<br>• URL of each funder website<br>• Did the sponsors or funders play any role in the study design, data collection and analysis, decision to publish, or preparation of the manuscript?<br><br>Did you receive funding for this work?	
Please add funding details.<br>as follow-up to "**Financial Disclosure**<br><br>Enter a financial disclosure statement that describes the sources of funding for the work included in this submission. Review the submission guidelines for detailed requirements. View published research articles from *PLOS ONE* for specific examples.<br><br>This statement is required for submission and **will appear in the published article** if the submission is accepted. Please make sure it is accurate.	This project was supported by a National Health and Medical Research Council (NHMRC) project grant (#1165936 to CGW, PHS, PF, DCW, PG, KC, AEC, MC) and NHMRC Synergy grant (#2009923 to PHS, PF, KC, AEC). AEC is supported by a NHMRC Investigator Grant (#2008454). www.nhmrc.gov.au/

**Funded studies**<br>Enter a statement with the following details:<br>• Initials of the authors who received each award<br>• Grant numbers awarded to each author<br>• The full name of each funder<br>• URL of each funder website<br>• Did the sponsors or funders play any role in the study design, data collection and analysis, decision to publish, or preparation of the manuscript?<br><br>Did you receive funding for this work?"	
Please select the country of your main research funder (please select carefully as in some cases this is used in fee calculation).<br>as follow-up to "**Financial Disclosure**<br><br>Enter a financial disclosure statement that describes the sources of funding for the work included in this submission. Review the submission guidelines for detailed requirements. View published research articles from *PLOS ONE* for specific examples.<br><br>This statement is required for submission and **will appear in the published article** if the submission is accepted. Please make sure it is accurate.<br><br>**Funded studies**<br>Enter a statement with the following details:<br>• Initials of the authors who received each award<br>• Grant numbers awarded to each author<br>• The full name of each funder<br>• URL of each funder website<br>• Did the sponsors or funders play any role in the study design, data collection and analysis, decision to publish, or preparation of the manuscript?<br><br>Did you receive funding for this work?"	AUSTRALIA - AU
**Competing Interests**	The authors have declared that no competing interests exist.

Use the instructions below to enter a competing interest statement for this submission. On behalf of all authors, disclose any competing interests that could be perceived to bias this work—acknowledging all financial support and any other relevant financial or non-financial competing interests.<br><br>This statement is required for submission and **will appear in the published article** if the submission is accepted. Please make sure it is accurate and that any funding sources listed in your Funding Information later in the submission form are also declared in your Financial Disclosure statement.<br><br>View published research articles from *PLOS ONE* for specific examples.<br><br>**NO authors have competing interests**<br><br>Enter: *The authors have declared that no competing interests exist.*<br><br>**Authors with competing interests**<br><br>Enter competing interest details beginning with this statement:<br><br>*I have read the journal's policy and the authors of this manuscript have the following competing interests: [insert competing interests here]*<br><br>* typeset	
**Ethics Statement**<br><br>Enter an ethics statement for this submission. This statement is required if the study involved:<br><br>• Human participants<br>• Human specimens or tissue<br>• Vertebrate animals or cephalopods<br>• Vertebrate embryos or tissues	The study has ethics approval from the NSW Population and Health Service Research Ethics Committee (2020/ETH03109).

Powered by Editorial Manager® and ProduXion Manager® from Aries Systems Corporation

• Field research<br><br>Write "N/A" if the submission does not require an ethics statement.<br><br>General guidance is provided below. Consult the submission guidelines for detailed instructions. **Make sure that all information entered here is included in the Methods section of the manuscript.**	

Powered by Editorial Manager® and ProduXion Manager® from Aries Systems Corporation

**Format for specific study types**<br><br>**Human Subject Research (involving human participants and/or tissue)**<br>• Give the name of the institutional review board or ethics committee that approved the study<br>• Include the approval number and/or a statement indicating approval of this research<br>• Indicate the form of consent obtained (written/oral) or the reason that consent was not obtained (e.g. the data were analyzed anonymously)<br><br>**Animal Research (involving vertebrate animals, embryos or tissues)**<br>• Provide the name of the Institutional Animal Care and Use Committee (IACUC) or other relevant ethics board that reviewed the study protocol, and indicate whether they approved this research or granted a formal waiver of ethical approval<br>• Include an approval number if one was obtained<br>• If the study involved *non-human primates*, add *additional details* about animal welfare and steps taken to ameliorate suffering<br>• If anesthesia, euthanasia, or any kind of animal sacrifice is part of the study, include briefly which substances and/or methods were applied<br><br>**Field Research**<br><br>Include the following details if this study involves the collection of plant, animal, or other materials from a natural setting:<br>• Field permit number<br>• Name of the institution or relevant body that granted permission	
**Data Availability**<br><br>Authors are required to make the data underlying their research findings fully available, without restriction.<br><br>If the manuscript reports pilot or preliminary results, the data underlying those results must be made available at the time of publication. A Data Availability	N/A - No results are reported

Statement describing where the data can be found is required at submission. Your answers to this question constitute the Data Availability Statement and will be published in the article, if accepted. When legal or ethical requirements prohibit public data sharing, authors must indicate how researchers may obtain access to the data. See the PLOS Data Policy and FAQ for detailed information.<br><br>**Important:** Stating 'data available on request from the author' is not sufficient. If your data are only available upon request, select 'No' for the first question and explain your situation in the text box.<br><br>For protocols without pilot or preliminary data, authors are strongly encouraged to state how they plan to share research data from their study when it is completed or published.<br><br>If your study includes pilot data, will they be made fully available without restriction?	
**Describe where the data may be found in full sentences. If you are copying our sample text, replace any instances of XXX with the appropriate details.**<br><br>• If **no pilot data are reported** in the manuscript, please enter the following: *No datasets were generated or analysed during the current study. All relevant data from this study will be made available upon study completion.*<br>• If the manuscript reports pilot data **held in a public repository**, include URLs, accession numbers or DOIs. If this information will only be available after acceptance, indicate this by ticking the box below. For example: *All XXX files are available from the XXX database (accession number(s) XXX, XXX.).*<br>• If the manuscript reports pilot data contained **within the manuscript and/or**	No datasets were generated or analysed during the current study. All relevant data from this study will be made available upon study completion, subject to ethics approval and data custodian approvals.

**Supporting Information files**, enter the following:<br>*All relevant data are within the manuscript and its Supporting Information files.*<br>• If neither of these applies but you are able to provide **details of access elsewhere**, with or without limitations, please do so. For example:<br><br>*Data cannot be shared publicly because of [XXX]. Data are available from the XXX Institutional Data Access / Ethics Committee (contact via XXX) for researchers who meet the criteria for access to confidential data.*<br><br>*The data underlying the results presented in the study are available from (include the name of the third party and contact information or URL).*<br>• This text is appropriate if the data are owned by a third party and authors do not have permission to share the data.<br><br>For study protocols with data management or sharing plans, authors are encouraged to briefly describe in their Data Availability Statement how data they generate will be made accessible when the study is completed. For example:<br>• Deidentified research data will be made publicly available when the study is completed and published.<br><br>* typeset	
Additional data availability information:	Tick here if your circumstances are not covered by the questions above and you need the journal's help to make your data available.

Manuscript

Click here to access/download;Manuscript;protocol_manuscript_20241028.do

A protocol for development of a microsimulation model platform to evaluate the potential benefits, harms, and cost-effectiveness of risk-tailored melanoma screening

Authors

Kirstie G McLoughlin[1], Caroline G Watts[1,2], Stephen Wade[1], Amelia K Smit[1,2], Peter H Soyer[3,4], Pablo Fernandez-Peñas[5,6], David C. Whiteman[7,8], Pascale Guitera[2,9,10], Gillian Reyes-Marcelino[1,2], Karen Canfell[1], Anne E Cust[1,2¶], Michael Caruana[1¶]

1. The Daffodil Centre, The University of Sydney, a joint venture with Cancer Council New South Wales, Sydney, New South Wales, Australia.
2. Melanoma Institute Australia, The University of Sydney, Sydney, New South Wales, Australia.
3. Frazer Institute, The University of Queensland, Dermatology Research Centre, Brisbane, Queensland, Australia.
4. Dermatology Department, Princess Alexandra Hospital, Brisbane, Queensland, Australia.
5. The University of Sydney, Faculty of Medicine and Health, Westmead Clinical School, NSW, Australia.
6. Department of Dermatology, Westmead Hospital, Westmead, New South Wales, Australia.
7. Departments of Population Health and Computational Biology, QIMR Berghofer Medical Research Institute, Queensland, Australia.
8. Faculty of Medicine, The University of Queensland, Queensland, Australia.
9. Faculty of Medicine and Health, The University of Sydney, Sydney, New South Wales, Australia.
10. Sydney Melanoma Diagnostic Centre, Royal Prince Alfred Hospital, New South Wales, Australia.

Corresponding author:

anne.cust@sydney.edu.au

[¶] AEC and MC are joint senior authors.

Abstract

In populations of European descent, melanoma is a high burden cancer in terms of incidence and healthcare costs, with early detection linked to better prognosis. There is no organised population screening program for melanoma in most countries, as more information is required about the potential benefits, harms and costs of population-based screening to develop policy. To assess the cost-effectiveness of potential risk-tailored organised melanoma screening programs, we have developed a protocol for a comprehensive microsimulation model (Policy1-Melanoma) that can evaluate multiple potential screening strategies. We outline the development of Policy1-Melanoma, a natural history model that will evaluate whether a risk-tailored organised melanoma screening program would be cost-effective in Australia. We specify the types of data sources used for calibration and validation of Policy1-Melanoma, and the steps in this process.

Keywords: melanoma, skin cancer, microsimulation, screening, surveillance, skin check, economic model, risk assessment, health decision modelling

Introduction

Melanoma accounts for nearly 2% of global cancer burden,[1] and its incidence globally has been steadily increasing.[2,3] Early diagnosis of melanoma is associated with high survival. This has driven interest in screening programs for melanoma, although the mortality benefit, cost-effectiveness, and potential harms from overdiagnosis (diagnosis of disease that would not have caused any symptoms or death) and overtreatment remains uncertain mainly due to a lack of randomised controlled trial data.[4] The US Preventive Services Task Force (USPSTF) has recently concluded that there is insufficient evidence to "assess the balance of benefits and harms of visual skin examination […] to screen for skin cancer".[4] An organised approach to screening for melanoma in line with the World Health Organisation's framework for population-based screening[5-7] has not been established at a national level in any country except for Germany.[8]

Whilst survival from late-stage melanoma has improved with new adjuvant therapies,[9] these treatments are very costly with later stage disease costing more than 60 times that of earlier stage disease in Australia.[10] Offsetting the growing costs of advanced cancer therapy could, in theory, improve the cost-effectiveness of screening approaches, as there are lower healthcare costs associated with early diagnosis. A better understanding of the benefits, harms and costs associated with structured approaches to the early detection of melanoma, including an organised national screening program, is needed to inform health policy. This is particularly true in Australia and New Zealand, where despite incidence rates in young people stabilising,[11] melanoma incidence remains the highest in the world.[3]

Microsimulation models are computer programs that simulate individual lifetimes and individual-specific events to model the possible impacts of various interventions. Individual-based simulations have the virtue of capturing heterogenous aspects of population risk, behaviour, and health care services implementation, as well as sex and histologically based differentials in risk, natural history, and outcomes. This is particularly important in the context of melanoma screening as lifetime events (such as

previous melanoma diagnosis or specific histological charact eristics) can increase or decrease the chance of future events (like diagnosis or melanoma-specific death). Such models are particularly useful in assessing the utility of public health interventions.[12,13] Given the difficulty, time, and cost of conducting large-scale screening trials with long-term follow-up, detailed mathematical models that simulate cancer burden in the population are often used as a valuable source of quantification in policy decision making. For example, the US Preventive Services Task Force has used models based on primary data from trials, contextualised in relation to local factors such as expected screening and management approaches and local burden of disease, to assess the utility of a range of approaches to cervical, breast, colorectal, and lung cancer screening.[14-17] Here we present a protocol for the development, calibration, and validation of a microsimulation platform to predict melanoma burden. We situate our melanoma model in the Australian population as an example context.

Methods and analysis

Natural history structure

Policy1-Melanoma is a new microsimulation platform in development from 2024-2026, using data inputs accessed from different sources including published data. Data used for model development were also obtained from the cancer registries for each state and territory in Australia, with ethics approval from the NSW Population and Health Service Research Ethics Committee (2020/ETH03109) and data accessed from 01/01/2022.

In collaboration with a multidisciplinary team, we have developed a natural history structure of melanoma that captures the key limiting epidemiological factors of this disease (Fig 1) and allows us to disentangle the effects of screening and surveillance. This discrete event microsimulation involves both pre- and post-diagnosis lifetime events (natural history and treatment pathways, respectively).

We model the development of first invasive or in situ melanomas by the three main melanoma histologies (superficial spreading, lentigo maligna, nodular) alongside an ‘other’ histology category that includes both unspecified and rarer subtypes, their diagnosis, and the potential for subsequent diagnoses of primary melanomas. These histopathological subtypes are incorporated into Policy1-Melanoma as they are associated with both the capacity for tumour detection and its growth and staging at diagnosis, and thus play an important role in understanding the potential utility of screening. For example, unlike superficial spreading and lentigo maligna melanomas that initially develop horizontally across the surface of the skin, nodular melanoma develops orthogonally to the skin and into the epidermis; this internally-directed growth means that nodular tumours are more difficult to visually detect at an early stage and are thus associated with a thicker Breslow thickness and later stage at diagnosis.[18] The progression through staging also varies between melanoma histologies; whilst a nodular melanoma might progress from in situ to stage 4 within a six-month period,[19] a lentigo maligna melanoma might make this progress over the course of 20 years.[20] The corollary of this differential growth is that screening is likely to detect melanomas that already have a better prognosis, a phenomenon known as length-time bias.[21]

Within Policy1-Melanoma, all individuals enter the simulation at age 15, in a ‘well’ state, with an associated risk of melanoma development and with no current skin check behaviour (Fig 1). Individuals can develop various melanoma states (both natural history and post-diagnosis) until they reach 85 or die, from melanoma-specific or other causes. Death from other causes can occur at any point in the simulation, but melanoma-specific deaths are only possible after a melanoma diagnosis. The assumptions underlying each transition within Policy1-Melanoma are summarised in Table 1.

Another characteristic related to melanoma incidence, which must be incorporated into the mathematical model, is the change in sun protection behaviours over time.[22-24] Reducing UV exposure, especially in childhood, is associated with lower risk of single and multiple primary melanoma.[25] Since 1980, Australia has implemented many public health programs to reduce sun exposure, which have been credited with reducing melanoma incidence in people under the age of 40 years.[26,27] To simulate the burden of

melanoma within Australia, birth-cohort variations must be considered. These preventative campaigns have also raised awareness of the early detection of melanoma and led to more prevalent opportunistic skin checks within the Australian population,[28] which act as non-standardised screening events. When assessing the utility of a melanoma screening program, the comparison group should be considered carefully given that opportunistic screening may be widespread.

Melanoma development within Policy1-Melanoma therefore depends upon age, sex, and birth cohort parameters. Combined, these factors provide a transition rate into one of four histologies, mentioned above. The effect of individual risk is to scale the ‘well’ to ‘undiagnosed melanoma’ transition rate. Specifically, all individuals have a probability of developing a melanoma, which is selected from a distribution which is affected by age, sex, and birth cohort. The effect of these factors is assumed to be constant between the risk groups, but the starting transition probability varies such that individuals in the high-risk group are more likely to develop melanomas at any time point. Whilst undiagnosed, the tumour can continue to develop. There are no distinct states for undiagnosed tumours, instead progression is implicitly modelled by assuming that the longer an individual remains undiagnosed the more likely they are to be diagnosed at a later stage and with worse survival-associated characteristics. Diagnosis is thus dependent upon time-since-melanoma-emergence (Fig 1), and the histological characteristics of the tumour.

Table 1. Assumptions and justifications of Policy1-Melanoma for calibration and/or future evaluations of cutaneous melanoma diagnosis and management in Australia

Transition	Assumptions	Justification and References
No skin checks to skin checks	In the context of population screening, screening usually refers to detection of a first primary cancer, thus individuals can only take up screening skin checks before their first primary melanoma is diagnosed.	After diagnosis of melanoma, skin checks form part of 'surveillance', not 'screening'. However, both screening and surveillance can be modelled.
	Uptake of skin checks depends on age, sex, individual risk level, and calendar year.	The proportion of the Australian population screening has increased over time.[28]
	Once skin checks are started, compliance may decline over time if there is no melanoma diagnosis.	Limited data available.
Risk groups	At the time of risk assessment, individuals remain in the same relative risk group throughout their lifetime or until first melanoma diagnosis.	Changes to potentially varying risk factors e.g. family history, non-melanoma skin cancer diagnosis, mole count, are not incorporated into the model. The impact of age is incorporated within the 'well' to 'undiagnosed first melanoma' transition.
	Individual risk level changes the probability of melanoma emerging, and associations of individual risk with melanoma incidence remain constant over time.	[29]
	Individual risk level changes the uptake of skin checks and adherence over time.	Individuals at higher risk are more likely to attend screening programs[30] and opportunistic skin checks.[28]
Well to undiagnosed first melanoma	First melanoma development depends on birth cohort, sex, individual risk level and age.	[29,31,32]
	Birth cohorts before 1972 have highest rates of melanoma development, between 1972 and 1992 rates decrease until the impact of sun protection campaigns is reached.	Sun protection behaviour campaigns in Australia started in 1982, particularly targeting children[24,26,33]. UV exposure influences melanoma risk more during childhood than adulthood.[34,35]
	There is no difference in the probability of a specific histological subtype diagnosis between the sexes.	Unpublished data.
	Relative proportion of histological subtypes has not changed over time, accounting for age.	Limited Australian data available.
	Individuals with any of the undiagnosed melanoma histologies have detectable melanoma. Before melanoma is detectable individuals remain "well".	
Undiagnosed first melanoma to diagnosis.	Attending a clinical skin check varies with patients' sex, age, individual risk level and time since melanoma development.<br>Detection by a clinician depends on the melanoma histological subtype, calendar period of diagnosis, time since melanoma development, and the body site of the melanoma.<br>Diagnosis depends on attending a clinical skin check and the ability of a clinician to detect a melanoma.	[28,36,37]
	Skin checks impact detection rates.	[38]

	Time left undiagnosed influences the stage at diagnosis.	[19,39,40]
	The speed and direction of melanoma growth depends on histological subtype.	[19]
Undiagnosed first melanoma to melanoma-specific death.	Breslow thickness and stage at diagnosis affect melanoma-specific survival. Within stage I and II, tumour thickness influences survival.	[9,41]
Lifetime surveillance.	Surveillance depends on stage at diagnosis.	[42]
Diagnosed melanoma (either first or subsequent) to undiagnosed subsequent melanoma.	The chance of subsequent primary diagnosis depends on individual risk level, histological subtype of first primary melanoma, and number of previous melanomas. Time to subsequent diagnosis of new primary melanoma decreases with number of previous primary melanomas.	[43,44]
	Subsequent primaries are more likely than not to be the same histological subtype as the first primary melanoma.	[45,46]
Undiagnosed subsequent primary melanoma to diagnosis.	Surveillance skin checks after a first diagnosis is a six-monthly or annual event.	[42,47]
	At a surveillance skin check the probability of a diagnosis depends upon the time since development and histological subtype of the tumour.	[36]
	For diagnosis of a subsequent primary melanoma, the same probability of attending a clinician and detection applies as for a first melanoma.	Limited data available.
Diagnosed subsequent melanoma to melanoma-specific death.	Melanoma-specific death can occur from any previous invasive melanoma (not necessarily the most recent).	[48]
	Stage at diagnosis and Breslow thickness influence survival.	[49] [9,41]

Factors influencing detection of melanoma

There are unique factors associated with melanoma that are different to many other cancer simulations, particularly that it is a cancer appearing on a visible surface (the skin), which impact the way we simulate melanoma development and diagnosis. The visibility of melanomas means that clinicians can, in theory, detect a melanoma incidentally with relative ease simply through seeing a patient during their normal practice.[36] The attendance of individuals to a doctor (such as a general practitioner) can, therefore, be expected to influence their rate of melanoma detection. For example, the slightly higher melanoma incidence in young women[50] may be partly an artefact of more frequent clinician interactions resulting in more detections in this age group rather than reflecting fewer extant cases in young men.

First melanoma diagnosis is thus affected by factors that influence clinician attendance (for example private health insurance and socioeconomic status) and also by factors that change the probability of detection once an individual is seen by a clinician; this is to capture the variation in clinician attendance between age and sex groups, the changes in detection technology over time, and the difference in difficulty diagnosing the specified melanoma histologies. Specifically, we note that whilst the likelihood of diagnosis of superficial spreading melanoma and lentigo maligna melanoma increase as they develop, there is not such an association for nodular melanomas due to their early and rapid vertical growth phase. Similar to the impact of risk upon melanoma emergence, the effect of routine skin checks upon diagnosis is implemented as a scaling factor upon the undiagnosed to diagnosed transition.

Diagnostic technologies have also improved, and usage has changed over time.[51,52] Dermoscopy has been shown to improve diagnostic sensitivity and reduce benign excisions among dermatologists and trained general practitioners.[53-56] Other technologies for diagnosing melanoma include total body photography incorporating 3D images, digital monitoring, in vivo reflectance confocal microscopy, and the use of machine learning algorithms.[57]

Once diagnosed, melanoma stage is defined depending upon the time-since emergence and tumour histology. There is no explicit stage progression after diagnosis - the potential for melanoma spread is incorporated implicitly into melanoma survival, alongside age, stage, and histological characteristics. Individuals who develop a first primary melanoma may develop a subsequent melanoma, die from advanced melanoma, or die from other causes (Fig 1).

Incorporation of personal risk and multiple primary melanomas

Another unique aspect of melanoma is that an individual can develop multiple primary melanomas over their lifetime, with risk of a new primary melanoma increasing with each new diagnosis,[44] although the number of melanomas developed does not necessarily worsen survival.[49] In Australia, the proportion of melanoma patients who develop a second primary melanoma over 10 years is about 16%,[58] although the risk varies according to the presence of other risk factors such as genetic predisposition.[44,59] After an initial invasive melanoma diagnosis patients usually remain under heightened lifetime skin surveillance, with annual or more frequent skin checks although guidelines do not usually make a distinction between invasive or in situ melanoma for skin check surveillance recommendations.[42,60] Australian guidelines for follow-up for risk of recurrence are based on stage at diagnosis, with more intensive follow-up for patients with greater risk of recurrence and the frequency of follow-up visits decreasing over time. [42,60] Long-term surveillance after melanoma diagnosis can inflate the costs associated with a screening program without providing additional benefits. Within Policy1-Melanoma, multiple primary melanomas are therefore incorporated separately.

The development of a subsequent primary melanoma, in Policy1-Melanoma, depends upon the histological subtype of the first primary melanoma's time since first diagnosed, as well as sex and personal risk level of the individual. The diagnosis of a subsequent primary melanoma is implemented as a surveillance event with diagnosis dependent upon tumour histology and patient history (including age,

number of previous primary diagnoses, and personal risk). We allow compliance to surveillance to decay based on time-since-diagnosis, histology, and stage at first diagnosis. We assume there is no difference in disease progression rates between a first and subsequent primary melanoma. Instead, progression rates and associated risk of development depend upon histological subtype for both first and subsequent melanomas. Implementing lifetime surveillance after a first melanoma diagnosis enables the separation of the effects of different surveillance methods and costs from that of the initial screening event.

Data inputs

There are many data inputs required for the microsimulation. A key input is age- and sex-specific melanoma incidence and mortality rates for first and subsequent primary melanomas from each of the eight Australian state and territory cancer registries, from 1982 to the latest available. Melanoma is a notifiable disease in Australia, and reporting is required for each jurisdiction. Tumours are classified using ICD-O-3 for body sites C44.0-C44.9 (and C80.9 melanoma of unknown primary site if not coded C44.9) and morphology codes M872-M879 with behaviour code 2 (in situ) or 3 (invasive). Primary care datasets[61,62] will be used to inform primary care clinician attendance patterns and the Medicare Benefits Schedule (a list of health professional services that the Australian Government subsidises)[63] for associated costs. Population-based studies including the Melanoma Patterns of Care Study[36] and National Sun Protection Survey[28] will be used to inform the patterns in skin check behaviours over time. Incidence rates of subsequent primary melanoma will be identified through an analysis of the cancer registry data and other population-based studies. Mortality rates will be incorporated into Policy1-Melanoma using relative survival, estimated from the cancer registries, and fitted against the reported mortality rates in Australia. The QSkin cohort study is a large skin cancer cohort in Queensland, Australia, that will be used for validation of the model.[64] Systematic reviews will be identified where relevant for other data inputs such as melanoma risk prediction tools[65] and health utilities for melanoma and other skin cancers.[66,67] Melanoma risk prediction tools will be used to stratify personal risk levels and incorporated into Policy1-

Melanoma to evaluate the potential impact of an organised risk-stratified melanoma screening program. Other transition probabilities will be informed through literature reviews or clinician elicitation.

Keratinocyte cancers and benign lesions will not be explicitly simulated in the initial iteration of the model. However, when evaluating the cost-effectiveness of risk-based melanoma screening programs, we will consider the financial implications of diagnosing these cancers and lesions in individuals participating in the program. Keratinocyte cancers and benign lesions will be incorporated into subsequent versions of the model.

Discussion

There are multiple applications to which Policy1-Melanoma can provide insights, including simulating risk-tailored screening, treatment, and surveillance pathways, quantifying the expected risk of overdiagnosis from screening and surveillance, and providing estimates on cost-effectiveness and resource implications of different pathways and on the impact of changing diagnostic and treatment options.

Given the inherent heterogeneity of melanoma risk, developing a model to assess the efficacy of a screening program necessitates a simulation that includes inter-person variability. Policy1-Melanoma conducts individual-based simulations, yet represents a simplification of the complex dynamics of melanoma growth in the Australian population. For example, many of the data inputs that contribute to the model development are overrepresented in people of European descent despite the increasingly ethnically diverse Australian population. Future iterations of screening models for Australia will need to incorporate the increasing heterogeneity of the Australian population.

Policy1-Melanoma will be used to provide evidence addressing the Australian Population Based Screening Framework[7] and Australian Cancer Plan[68] so that the Australian Government, and ultimately

other countries, can assess the merits of a potential organised, risk-stratified, melanoma national screening program, which would sit alongside already established screening programs for breast, bowel, and cervical cancers.

Acknowledgements: We would like to thank the Investigators of the Melanoma Screening Modelling and Implementation Project for their broad contributions to this research program.

References

1. Bray F, Ferlay J, Soerjomataram I, et al. Global cancer statistics 2018: GLOBOCAN estimates of incidence and mortality worldwide for 36 cancers in 185 countries. *CA Cancer J Clin* 2018;68(6):394-424. doi: 10.3322/caac.21492

2. Erdmann F, Lortet-Tieulent J, Schuz J, et al. International trends in the incidence of malignant melanoma 1953-2008--are recent generations at higher or lower risk? *Int J Cancer* 2013;132(2):385-400. doi: 10.1002/ijc.27616

3. Arnold M, Singh D, Laversanne M, et al. Global Burden of Cutaneous Melanoma in 2020 and Projections to 2040. *JAMA Dermatol* 2022;158(5):495-503. doi: 10.1001/jamadermatol.2022.0160

4. US Preventive Services Task Force, Mangione CM, Barry MJ, et al. Screening for Skin Cancer: US Preventive Services Task Force Recommendation Statement. *JAMA* 2023;329(15):1290-95. doi: 10.1001/jama.2023.4342

5. Wilson JM, Jungner YG. [Principles and practice of mass screening for disease]. *Bol Oficina Sanit Panam* 1968;65(4):281-393.

6. Dobrow MJ, Hagens V, Chafe R, et al. Consolidated principles for screening based on a systematic review and consensus process. *CMAJ* 2018;190(14):E422-E29. doi: 10.1503/cmaj.171154

7. Population Based Screening Framework, Commonwealth of Australia (Department of Health), Canberra, 2018.

8. Datzmann T, Schoffer O, Meier F, et al. Are patients benefiting from participation in the German skin cancer screening programme? A large cohort study based on administrative data. *Br J Dermatol* 2022;186(1):69-77. doi: 10.1111/bjd.20658

9. Gershenwald JE, Scolyer RA, Hess KR, et al. Melanoma staging: Evidence-based changes in the American Joint Committee on Cancer eighth edition cancer staging manual. *CA Cancer J Clin* 2017;67(6):472-92. doi: 10.3322/caac.21409

10. Elliott TM, Whiteman DC, Olsen CM, et al. Estimated Healthcare Costs of Melanoma in Australia Over 3 Years Post-Diagnosis. *Appl Health Econ Health Policy* 2017;15(6):805-16. doi: 10.1007/s40258-017-0341-y

11. Venugopal K, Youlden D, Marvelde LT, et al. Twenty years of melanoma in Victoria, Queensland, and South Australia (1997 - 2016). *Cancer Epidemiol* 2023;83:102321. doi: 10.1016/j.canep.2023.102321

12. Rutter CM, Zaslavsky AM, Feuer EJ. Dynamic microsimulation models for health outcomes: a review. *Med Decis Making* 2011;31(1):10-8. doi: 10.1177/0272989X10369005

13. Lew JB, Feletto E, Wade S, et al. Benefits, harms and cost-effectiveness of cancer screening in Australia: an overview of modelling estimates. *Public Health Res Pract* 2019;29(2) doi: 10.17061/phrp2921913

14. US Preventive Services Task Force, Curry SJ, Krist AH, et al. Screening for Cervical Cancer: US Preventive Services Task Force Recommendation Statement. *JAMA* 2018;320(7):674-86. doi: 10.1001/jama.2018.10897

15. US Preventive Services Task Force, Davidson KW, Barry MJ, et al. Screening for Colorectal Cancer: US Preventive Services Task Force Recommendation Statement. *JAMA* 2021;325(19):1965-77. doi: 10.1001/jama.2021.6238

16. US Preventive Services Task Force, Krist AH, Davidson KW, et al. Screening for Lung Cancer: US Preventive Services Task Force Recommendation Statement. *JAMA* 2021;325(10):962-70. doi: 10.1001/jama.2021.1117

17. Siu AL, Force USPST. Screening for Breast Cancer: U.S. Preventive Services Task Force Recommendation Statement. *Ann Intern Med* 2016;164(4):279-96. doi: 10.7326/M15-2886

18. Mar V, Roberts H, Wolfe R, et al. Nodular melanoma: a distinct clinical entity and the largest contributor to melanoma deaths in Victoria, Australia. *J Am Acad Dermatol* 2013;68(4):568-75. doi: 10.1016/j.jaad.2012.09.047

19. Liu W, Dowling JP, Murray WK, et al. Rate of growth in melanomas: characteristics and associations of rapidly growing melanomas. *Arch Dermatol* 2006;142(12):1551-8. doi: 10.1001/archderm.142.12.1551

20. Menzies SW, Liyanarachchi S, Coates E, et al. Estimated risk of progression of lentigo maligna to lentigo maligna melanoma. *Melanoma Res* 2020;30(2):193-97. doi: 10.1097/CMR.0000000000000619

21. Jacklyn G, Bell K, Hayen A. Assessing the efficacy of cancer screening. *Public Health Res Pract* 2017;27(3) doi: 10.17061/phrp2731727

22. Tabbakh T, Volkov A, Wakefield M, et al. Implementation of the SunSmart program and population sun protection behaviour in Melbourne, Australia: Results from cross-sectional summer surveys from 1987 to 2017. *PLoS Med* 2019;16(10):e1002932. doi: 10.1371/journal.pmed.1002932

23. Dobbinson SJ, Wakefield MA, Jamsen KM, et al. Weekend sun protection and sunburn in Australia trends (1987-2002) and association with SunSmart television advertising. *Am J Prev Med* 2008;34(2):94-101. doi: 10.1016/j.amepre.2007.09.024

24. Hunkin H, Pollock K, Scupham R. The National SunSmart Schools Program: Impact on sun protection policies and practices in Australian primary schools. *Health Promot J Austr* 2020;31(2):251-57. doi: 10.1002/hpja.291

25. Kricker A, Armstrong BK, Goumas C, et al. Ambient UV, personal sun exposure and risk of multiple primary melanomas. *Cancer Causes Control* 2007;18(3):295-304. doi: 10.1007/s10552-006-0091-x

26. Walker H, Maitland C, Tabbakh T, et al. Forty years of Slip! Slop! Slap! A call to action on skin cancer prevention for Australia. *Public Health Res Pract* 2022;32(1) doi: 10.17061/phrp31452117

27. Olsen CM, Green AC, Pandeya N, et al. Trends in Melanoma Incidence Rates in Eight Susceptible Populations through 2015. *J Invest Dermatol* 2019;139(6):1392-95. doi: 10.1016/j.jid.2018.12.006

28. Reyes-Marcelino G, Tabbakh T, Espinoza D, et al. Prevalence of skin examination behaviours among Australians over time. *Cancer Epidemiol* 2021;70:101874. doi: 10.1016/j.canep.2020.101874

29. Olsen CM, Pandeya N, Thompson BS, et al. Risk Stratification for Melanoma: Models Derived and Validated in a Purpose-Designed Prospective Cohort. *J Natl Cancer Inst* 2018;110(10):1075-83. doi: 10.1093/jnci/djy023

30. Koh HK, Norton LA, Geller AC, et al. Evaluation of the American Academy of Dermatology's National Skin Cancer Early Detection and Screening Program. *J Am Acad Dermatol* 1996;34(6):971-8. doi: 10.1016/s0190-9622(96)90274-1

31. Aitken JF, Youlden DR, Baade PD, et al. Generational shift in melanoma incidence and mortality in Queensland, Australia, 1995-2014. *Int J Cancer* 2018;142(8):1528-35. doi: 10.1002/ijc.31141

32. Whiteman DC, Green AC, Olsen CM. The Growing Burden of Invasive Melanoma: Projections of Incidence Rates and Numbers of New Cases in Six Susceptible Populations through 2031. *J Invest Dermatol* 2016;136(6):1161-71. doi: 10.1016/j.jid.2016.01.035

33. Reyes-Marcelino G, Wang R, Gultekin S, et al. School-based interventions to improve sun-safe knowledge, attitudes and behaviors in childhood and adolescence: A systematic review. *Prev Med* 2021;146:106459. doi: 10.1016/j.ypmed.2021.106459

34. Whiteman DC, Whiteman CA, Green AC. Childhood sun exposure as a risk factor for melanoma: a systematic review of epidemiologic studies. *Cancer Causes Control* 2001;12(1):69-82. doi: 10.1023/a:1008980919928

35. Cust AE, Armstrong BK, Goumas C, et al. Sunbed use during adolescence and early adulthood is associated with increased risk of early-onset melanoma. *Int J Cancer* 2011;128(10):2425-35. doi: 10.1002/ijc.25576

36. Watts CG, McLoughlin K, Goumas C, et al. Association Between Melanoma Detected During Routine Skin Checks and Mortality. *JAMA Dermatol* 2021;157(12):1425-36. doi: 10.1001/jamadermatol.2021.3884

37. Watts CG, Madronio C, Morton RL, et al. Clinical Features Associated With Individuals at Higher Risk of Melanoma: A Population-Based Study. *JAMA Dermatol* 2017;153(1):23-29. doi: 10.1001/jamadermatol.2016.3327

38. Whiteman DC, Olsen CM, MacGregor S, et al. The effect of screening on melanoma incidence and biopsy rates. *Br J Dermatol* 2022;187(4):515-22. doi: 10.1111/bjd.21649

39. Lin MJ, Mar V, McLean C, et al. An objective measure of growth rate using partial biopsy specimens of melanomas that were initially misdiagnosed. *J Am Acad Dermatol* 2014;71(4):691-7. doi: 10.1016/j.jaad.2014.04.068

40. Paul E, Pausch A, Bodeker RH. Speed of growth of melanoma: statistical analysis of the average ages of patient groups. *Pigment Cell Res* 1989;2(6):475-7. doi: 10.1111/j.1600-0749.1989.tb00241.x

41. Baade PD, Whiteman DC, Janda M, et al. Long-term deaths from melanoma according to tumor thickness at diagnosis. *Int J Cancer* 2020;147(5):1391-96. doi: 10.1002/ijc.32930

42. Barbour AG, A; Liu,W; Menzies,S; Morton,R; Cancer Council Australia Melanoma Guidelines Working Party,. Follow up after initial definitive treatment for each stage of melanoma 2018 [Available from: https://app.magicapp.org/#/guideline/Lkk3pL2024.

43. Smith J, Cust AE, Lo SN. Risk factors for subsequent primary melanoma in patients with previous melanoma: a systematic review and meta-analysis. *Br J Dermatol* 2024;190(2):174-83. doi: 10.1093/bjd/ljad275

44. Cust AE, Badcock C, Smith J, et al. A risk prediction model for the development of subsequent primary melanoma in a population-based cohort. *Br J Dermatol* 2020;182(5):1148-57. doi: 10.1111/bjd.18524

45. Murali R, Goumas C, Kricker A, et al. Clinicopathologic features of incident and subsequent tumors in patients with multiple primary cutaneous melanomas. *Ann Surg Oncol* 2012;19(3):1024-33. doi: 10.1245/s10434-011-2058-8

46. Scheibner A, Milton GW, McCarthy WH, et al. Multiple primary melanoma - a review of 90 cases. *Australas J Dermatol* 1982;23(1):1-8. doi: 10.1111/j.1440-0960.1982.tb00230.x

47. Mann GC, Anne; Damian, Diona; Fishburn, Paul; Kelly, John; Mar, Victoria; Soyer, H. Peter; Cancer Council Australia Melanoma Guidelines Working Party. What interventions have been shown to provide clinical benefit in those assessed to be at high risk of new primary melanoma? 2018 [Available from: https://app.magicapp.org/#/guideline/Lkk3pL2024.

48. Peek G, Olsen CM, Baade P, et al. Survival in patients with multiple primary melanomas: Systematic review and meta-analysis. *J Am Acad Dermatol* 2020;83(5):1406-14. doi: 10.1016/j.jaad.2020.03.076

49. Ni Y, Watts CG, Scolyer RA, et al. Prognosis for people with multiple primary melanomas compared with a single primary melanoma. *J Am Acad Dermatol* 2023 doi: 10.1016/j.jaad.2023.08.089

50. Iannacone MR, Youlden DR, Baade PD, et al. Melanoma incidence trends and survival in adolescents and young adults in Queensland, Australia. *Int J Cancer* 2015;136(3):603-9. doi: 10.1002/ijc.28956

51. Garrison ZR, Hall CM, Fey RM, et al. Advances in Early Detection of Melanoma and the Future of At-Home Testing. *Life (Basel)* 2023;13(4) doi: 10.3390/life13040974

52. Bhave P, Wong J, McInerney-Leo A, et al. Management of cutaneous melanoma in Australia: a narrative review. *Med J Aust* 2023;218(9):426-31. doi: 10.5694/mja2.51910

53. Herschorn A. Dermoscopy for melanoma detection in family practice. *Can Fam Physician* 2012;58(7):740-5, e372-8.

54. Holmes GA, Vassantachart JM, Limone BA, et al. Using Dermoscopy to Identify Melanoma and Improve Diagnostic Discrimination. *Fed Pract* 2018;35(Suppl 4):S39-S45.

55. Argenziano G, Puig S, Zalaudek I, et al. Dermoscopy improves accuracy of primary care physicians to triage lesions suggestive of skin cancer. *J Clin Oncol* 2006;24(12):1877-82. doi: 10.1200/JCO.2005.05.0864

56. Menzies SW, Emery J, Staples M, et al. Impact of dermoscopy and short-term sequential digital dermoscopy imaging for the management of pigmented lesions in primary care: a sequential intervention trial. *Br J Dermatol* 2009;161(6):1270-7. doi: 10.1111/j.1365-2133.2009.09374.x

57. Grossarth S, Mosley D, Madden C, et al. Recent Advances in Melanoma Diagnosis and Prognosis Using Machine Learning Methods. *Current oncology reports* 2023;25(6):635-45. doi: 10.1007/s11912-023-01407-3

58. Ni Y, Watts CG, Scolyer RA, et al. Risk of developing a second primary melanoma after a first primary melanoma in a population-based Australian cohort. *Br J Dermatol* 2023;188(6):814-16. doi: 10.1093/bjd/ljad076

59. Olsen CM, Pandeya N, Dusingize JC, et al. Risk Factors Associated With First and Second Primary Melanomas in a High-Incidence Population. *JAMA Dermatol* 2023;159(1):37-46. doi: 10.1001/jamadermatol.2022.4975

60. Read RL, Madronio CM, Cust AE, et al. Follow-Up Recommendations after Diagnosis of Primary Cutaneous Melanoma: A Population-Based Study in New South Wales, Australia. *Ann Surg Oncol* 2018;25(3):617-25. doi: 10.1245/s10434-017-6319-z

61. So C, McCarthy D, Watts CG, et al. Diagnostic biopsies of suspected skin cancer in general practice from 2010 to 2017 in Victoria, Australia. *Br J Dermatol* 2023;188(4):560-61. doi: 10.1093/bjd/ljac111

62. Reyes-Marcelino G, McLoughlin K, Harrison C, et al. Skin cancer-related conditions managed in general practice in Australia, 2000-2016: a nationally representative, cross-sectional survey. *BMJ Open* 2023;13(5):e067744. doi: 10.1136/bmjopen-2022-067744

63. Commonwealth of Australia, Department of Health and Aged Care: https://www.health.gov.au/our-work/mbs-review; 2023 [accessed 28/03/2024.

64. Olsen CM, Green AC, Neale RE, et al. Cohort profile: the QSkin Sun and Health Study. *Int J Epidemiol* 2012;41(4):929-29i. doi: 10.1093/ije/dys107

65. Kaiser I, Pfahlberg AB, Uter W, et al. Risk Prediction Models for Melanoma: A Systematic Review on the Heterogeneity in Model Development and Validation. *Int J Environ Res Public Health* 2020;17(21) doi: 10.3390/ijerph17217919

66. So C, Cust AE, Gordon LG, et al. Health utilities for non-melanoma skin cancers and pre-cancerous lesions: A systematic review. *Skin Health Dis* 2021;1(3):e51. doi: 10.1002/ski2.51

67. Dieng M, Kasparian NA, Cust AE, et al. Sensitivity of Preference-Based Quality-of-Life Measures for Economic Evaluations in Early-Stage Melanoma. *JAMA Dermatol* 2018;154(1):52-59. doi: 10.1001/jamadermatol.2017.4701

68. Cancer Australia. Australian Cancer Plan. Surry Hills, NSW, 2023.

Data availability statement: There are no data available as yet for Policy1-Melanoma.

Authors' contributions:

Conceptualization: KGM, CGW, PHS, PF, DCW, PG, KC, AEC, MC

Data curation: KGM, SW, MC

Formal Analysis: KGM, MC

Funding acquisition: CW, PHS, PF, DCW, PG, KC, AEC, MC

Investigation: KGM, CGW, AKS, GR, KC, AEC, MC

Methodology: KGM, CGW, SW, PHS, PF, DCW, PG, KC, AEC, MC

Project administration: KGM, KC, AEC, MC

Resources: KC, AEC, MC

Software: KGM, KC, MC

Supervision: KC, AEC, MC

Validation: KGM, CGW, AKS, KC, AEC, MC

Visualization: KGM, CGW, AKS, KC, AEC, MC

Writing – original draft: KGM, CGW, AEC, MC

Writing – review & editing: KGM, CGW, SW, AKS, PHS, PF, DCW, PG, GR, KC, AEC, MC

Competing interests statement: There are no conflicts of interest to declare.

Figure legends:

Fig 1. Schematic of the Policy1-Melanoma model structure. Individuals enter the simulation at age 15 in a healthy ('well') state having previously undertaken no routine skin checks. Over the course of their lifetime, they may develop a first primary melanoma with one of three specific histological subtypes or no specified histological subtype. At any point before diagnosis an individual can take up routine skin checks, which changes the probability of an undiagnosed melanoma being identified (dotted line). Melanoma diagnosis rates depend upon the sex, age, and skin check behaviour of the individual, their tumour histological subtype, and the period in which the identifying interaction (clinician visit) occurs. Once a first primary melanoma is diagnosed, a stage is allocated depending on tumour histological subtype and the time between its development and diagnosis. The individual may die of their melanoma, where survival is stage dependent. Most individuals do not die of their first melanoma and therefore have the potential to develop a secondary (or subsequent) primary melanoma. The probability of subsequent melanoma diagnosis is based on lifetime surveillance.

Figure 1

Click here to access/download;Figure;Figure 1.jpg

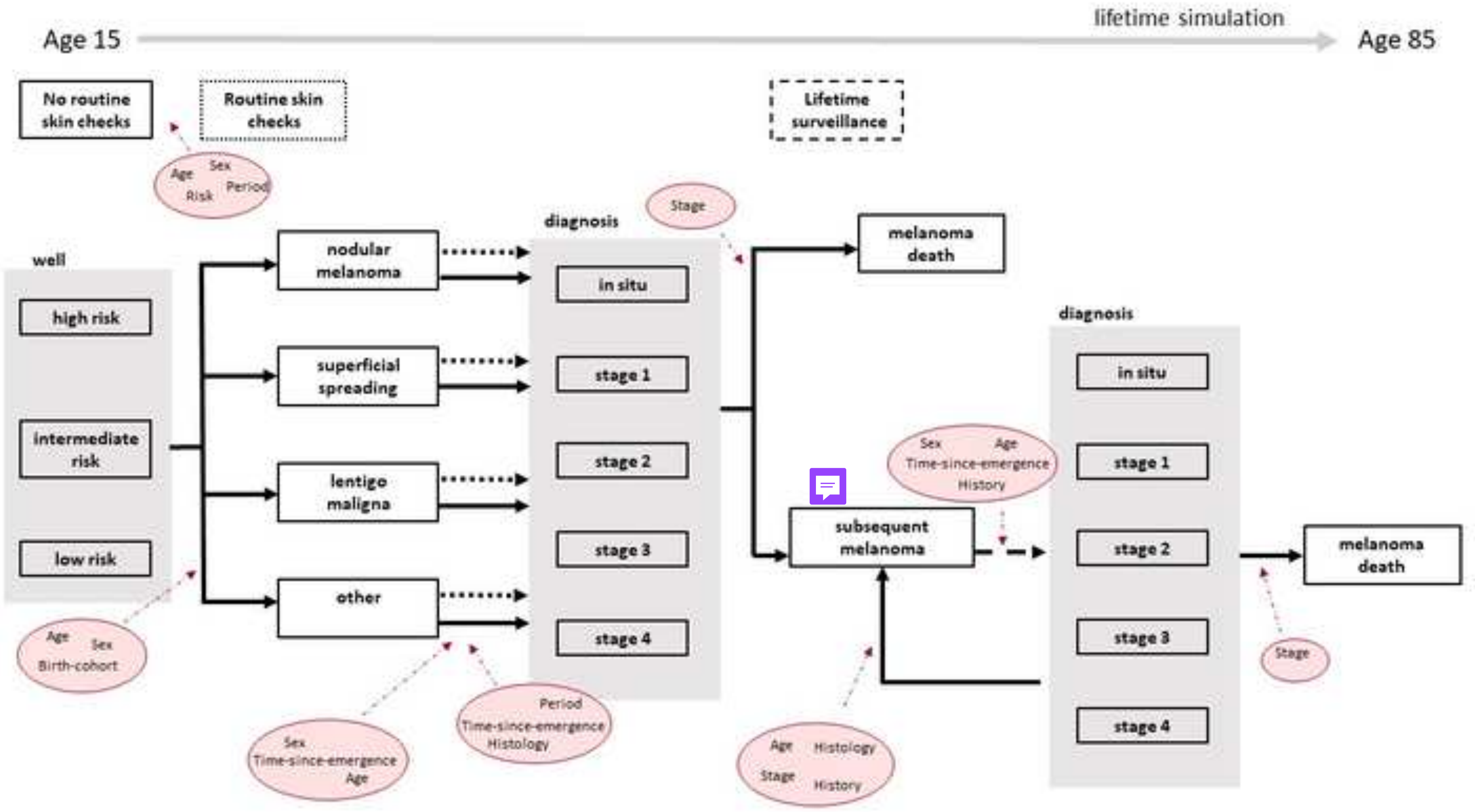

Supplement: Attachment — Submitted filename: PONE-D-24-40002_reviewer.pdf [file pone.0339177.s002.pdf]
