# Supplementary material for: A protocol for development of a microsimulation model platform to evaluate the potential benefits, harms, and cost-effectiveness of risk-tailored melanoma screening
Source: PLoS One. 2025 Dec 26;20(12):e0339177. doi: 10.1371/journal.pone.0339177 (PMC12742747; doi:10.1371/journal.pone.0339177)
Supplement: Attachment — Submitted filename: Responses_to_reviewers_20250122.docx [file pone.0339177.s005.docx]

**Journal Requirements:**

1. Please ensure that your manuscript meets PLOS ONE's style requirements, including those for file naming.

Author response: We comply with the PLOS ONE style requirements.

2. Thank you for stating the following financial disclosure:

"This project was supported by a National Health and Medical Research Council (NHMRC) project grant (#1165936 to CGW, PHS, PF, DCW, PG, KC, AEC, MC) and NHMRC Synergy grant (#2009923 to PHS, PF, KC, AEC). AEC is supported by a NHMRC Investigator Grant (#2008454). [www.nhmrc.gov.au/](http://www.nhmrc.gov.au/)"

Author response: We have added this financial disclosure on page 14 immediately before the References.

Please state what role the funders took in the study.  If the funders had no role, please state: ""The funders had no role in study design, data collection and analysis, decision to publish, or preparation of the manuscript.""

If this statement is not correct you must amend it as needed.

Please include this amended Role of Funder statement in your cover letter; we will change the online submission form on your behalf.

Author response: We have added this sentence to the financial disclosure on page 14 and to the cover letter.

**Reviewers' comments:**
Reviewer's Responses to Questions

**Comments to the Author**
1. Does the manuscript provide a valid rationale for the proposed study, with clearly identified and justified research questions?
The research question outlined is expected to address a valid academic problem or topic and contribute to the base of knowledge in the field.

Reviewer #1: Yes

Reviewer #2: Yes

2. Is the protocol technically sound and planned in a manner that will lead to a meaningful outcome and allow testing the stated hypotheses?
The manuscript should describe the methods in sufficient detail to prevent undisclosed flexibility in the experimental procedure or analysis pipeline, including sufficient outcome-neutral conditions (e.g. necessary controls, absence of floor or ceiling effects) to test the proposed hypotheses and a statistical power analysis where applicable. As there may be aspects of the methodology and analysis which can only be refined once the work is undertaken, authors should outline potential assumptions and explicitly describe what aspects of the proposed analyses, if any, are exploratory.

Reviewer #1: Partly

Reviewer #2: Yes

3. Is the methodology feasible and described in sufficient detail to allow the work to be replicable?
Descriptions of methods and materials in the protocol should be reported in sufficient detail for another researcher to reproduce all experiments and analyses. The protocol should describe the appropriate controls, sample size calculations, and replication needed to ensure that the data are robust and reproducible.

Reviewer #1: Yes

Reviewer #2: No

4. Have the authors described where all data underlying the findings will be made available when the study is complete?
The [PLOS Data policy](https://aus01.safelinks.protection.outlook.com/?url=https%3A%2F%2Furl.au.m.mimecastprotect.com%2Fs%2F2mm1C1WLPxcEKRP4OU1uvIVOC1B%3Fdomain%3Dtrack.editorialmanager.com&data=05%7C02%7CKirstie.McLoughlin%40nswcc.org.au%7Cb2fe1fb2cb26453720af08dd1567724e%7Cd1915bf415464990bcd4abdb03b89703%7C0%7C0%7C638690256700638718%7CUnknown%7CTWFpbGZsb3d8eyJFbXB0eU1hcGkiOnRydWUsIlYiOiIwLjAuMDAwMCIsIlAiOiJXaW4zMiIsIkFOIjoiTWFpbCIsIldUIjoyfQ%3D%3D%7C0%7C%7C%7C&sdata=tWMqlc4ei5KE4tmLzBMuEFqhbD7dxEvpGZzFXwgW1EI%3D&reserved=0) requires authors to make all data underlying the findings described in their manuscript fully available without restriction, with rare exception, at the time of publication. The data should be provided as part of the manuscript or its supporting information, or deposited to a public repository. For example, in addition to summary statistics, the data points behind means, medians and variance measures should be available. If there are restrictions on publicly sharing data—e.g. participant privacy or use of data from a third party—those must be specified.

Reviewer #1: Yes

Reviewer #2: No

5. Is the manuscript presented in an intelligible fashion and written in standard English?
PLOS ONE does not copyedit accepted manuscripts, so the language in submitted articles must be clear, correct, and unambiguous. Any typographical or grammatical errors should be corrected at revision, so please note any specific errors here.

Reviewer #1: Yes

Reviewer #2: Yes

**6. Review Comments to the Author**
Please use the space provided to explain your answers to the questions above and, if applicable, provide comments about issues authors must address before this protocol can be accepted for publication. You may also include additional comments for the author, including concerns about research or publication ethics.
You may also provide optional suggestions and comments to authors that they might find helpful in planning their study.
(Please upload your review as an attachment if it exceeds 20,000 characters)

**Reviewer #1:**

Dear all,
Thank you for providing me with the opportunity of reviewing this nice protocol paper for the development of a health economic model. I would really like to congratulate the authors on taking up this initiative since I think we, as a field, take too little time to write such protocols for the development of such models, while they are a valuable tool to think about structural assumptions of a model and potentially prevent duplication of work. I think this type of initiatives should be praised within the health economics and outcomes research field.
Please find in the attached PDF file my detailed comments on the text.

Author response: Thank you Prof. Pouwels for attaching your detailed comments marked on the manuscript file. We have gone through each comment and made changes in the text in response. We have also responded to your specific comments below.

My main comments are the following:
1. The authors mention in their manuscript that they will develop a discrete event simulation. However they do not provide any information on which time-to-event distributions will be considered and how competing events will be addressed within the health economic model. Please add a section on these issues.

Author response:

We have added the following details to the text (page 5): “Competing events within Policy1-Melanoma will be dealt with using event-specific distributions; the time at which each competing event occurs is predicted based on transition probabilities and the earliest occurring event is selected.^22^”

2. The authors describe certain aspects of melanoma screening in quite general terms and do not always relate this information to how it will be included in the health economic model, please be more specific on how the information mentioned in the manuscript informs structural assumptions or inputs of the health economic model.

Author response:

We have made edits in the manuscript to address this, for example page 9-10 where we describe how the effect of changing diagnostic technologies is incorporated into the model: “The effect of changing diagnostic techniques is incorporated into Policy1-Melanoma as a scaling function, improving diagnostic rates until they match observed diagnostic technology usage in Australia. Screening within Policy1-Melanoma is implemented as a detection event. Individuals ‘attend’ a screening event at which, if they have an undiagnosed melanoma, melanoma can be diagnosed. The probability of melanoma being diagnosed at a screening event depends upon the stage, histology, and location of the undiagnosed tumour. The improved diagnostic rates from dermoscopy (or other diagnostic technologies) are also included as period effects in screening detection probability.”

We note that the modelling platform will be able to be used to investigate many scenarios within different populations. We hope that by designing a simulation which is more generalisable than standard health economic models we will be able to use the platform to answer policy questions which have not yet arisen.

3. I would like to suggest to structure the text of this protocol according to the “health economics analysis plan” described in Thorn et al. since I think not all relevant aspects of the model are equally thoroughly mentioned (Thorn JC, Davies CF, Brookes ST, Noble SM, Dritsaki M, Gray E, Hughes DA, Mihaylova B, Petrou S, Ridyard C, Sach T. Content of Health Economics Analysis Plans (HEAPs) for trial-based economic evaluations: expert Delphi consensus survey. Value in Health. 2021 Apr 1;24(4):539-47.). Even though this reference seems to focus on trial-based economic evaluations, it also contains a section dedicated to health economic models, and of course the general sections apply to this protocol as well. Good luck with updating this manuscript! Xavier Pouwels

Author response:

Thank you for this suggestion. We agree that it is important that models are reported clearly, and we have made changes to the manuscript throughout to improve its clarity. For example, we have now included a new section included on ‘Costs of melanoma’ (page 13 before the Discussion), and more details in the ‘Data inputs’ section. We show the proposed checklist from Thorn et al (2021) below, and indicate where the relevant information is contained within the manuscript. Where information was missing, this has now been added to the manuscript. If an included component is missing from the manuscript we have indicated why it has not been incorporated or is not relevant. Whilst we have incorporated additional components into the text, our preference is to retain our current manuscript structure, as this enables us to better highlight the conceptual uniqueness of melanoma simulations (e.g. as a visible, high-survival, potentially repeatable cancer) when compared to simulations of other cancer types.

Thorn et al (2021) expected content of health economic analysis plans.

| **Item** | **Item status after final voting** | **Item included in protocol?** |
| --- | --- | --- |
| Title | IN | Yes. |
| Trial registration number | IN | NA – not a trial |
| Source of funding | IN | Yes - added |
| Purpose of health economics analysis plan (HEAP) | IN | NA –not a trial, but the Aims of the protocol are specified in the Abstract and Introduction |
| Sponsor approval | OUT | No |
| Trial protocol version | IN | NA – not a trial |
| Trial statistical analysis plan (SAP) version | IN | NA – not a trial |
| Trial HEAP version | IN | NA – not a trial |
| HEAP revisions | IN | NA – not a trial |
| Table of contents | OPTIONAL LIST | No |
| Abbreviations/glossary of terms/definitions | OPTIONAL LIST | No |
| Roles and responsibilities | IN | Yes. See Authors Contributions section. |
| Signature(s) of person(s) writing HEAP (and date) | IN | NA – not a trial |
| Signature of senior health economist (HE) who is guarantor of the economic evaluation (and date) | IN | NA – not a trial |
| Signature of the chief investigator for the trial | IN | NA – not a trial |
| Trial background and rationale | IN | NA. Background information in Introduction. |
| Aim(s) of the trial | IN | NA – this is not a trial, but the Aims of the protocol are specified in the Abstract and Introduction |
| Objectives and/or research hypotheses of the trial | IN | NA as above |
| Trial population | IN | Yes. See Introduction & Methods and analysis. |
| Intervention and comparator(s) | IN | NA. Protocol is for a simulation which can be used in multiple contexts. |
| Trial design | IN | NA – not a trial |
| Trial start and end dates | IN | NA – not a trial |
| Aim(s) of economic evaluation | IN | This is a protocol for a generalisable microsimulation model of melanoma that can be flexibly used to evaluate a range of scenarios related to melanoma screening, diagnosis, surveillance and management (rather than a specific economic evaluation). However, in the Abstract and Introduction we mention our specific aim to evaluate skin cancer screening. |
| Objectives(s)/hypotheses of economic evaluation | IN | As above |
| Overview of economic analysis | IN | Methods and Analysis |
| Jurisdiction | IN | Yes. See Introduction & Methods and analysis. |
| Perspective(s) | IN | Yes – see Costs of melanoma section. |
| Time horizon | IN | Yes – added lifetime time horizon. See Methods and analysis section – Natural history structure. |
| Monitoring collection of health economic data | OPTIONAL LIST | No. |
| Database management | OPTIONAL LIST | NA. |
| Data entry | OPTIONAL LIST | NA |
| Data cleaning for analysis | IN | No. Data was cleaned by the Australian Cancer Registries. Additional cleaning may be performed before economic analyses are performed and this information would be provided in the context of a published model and specific evaluations. |
| Data archiving | OPTIONAL LIST | NA |
| Statistical software used for HE analysis | IN | Yes - added. See Methods and analysis section. |
| Identification of resources | IN | Yes – see Costs of melanoma section. |
| Measurement of resource use data | IN | Yes - as above |
| Valuation of resource use data | IN | Yes - see Costs of melanoma section. |
| Identification of outcome(s) | IN | Yes - see Costs of melanoma section. |
| Measurement of outcome(s) | IN | Yes - see Costs of melanoma section. More details will be provided in later manuscripts showing results of specific evaluations. |
| Valuation of outcome(s) | IN | Yes - see Costs of melanoma section. More details will be provided in later manuscripts showing results of specific evaluations. |
| Analysis population | IN | See Methods and Analysis for broad population details |
| Timing of analyses | IN | No – as the analyses will be ongoing |
| Discount rates for costs and benefits | IN | Yes - see Costs of melanoma section. |
| Cost-effectiveness threshold(s) | IN | Yes - see Costs of melanoma section. |
| Statistical decision rule(s) | IN | NA – not a trial, will depend on specific evaluation |
| Analysis of resource use | IN | NA – not a trial, will depend on specific evaluation |
| Analysis of costs | IN | NA – not a trial, will depend on specific evaluation |
| Analysis of outcomes | IN | NA – not a trial, will depend on specific evaluation |
| Missing data | IN | NA – not a trial, will depend on specific evaluation |
| Analysis of cost-effectiveness | IN | Yes – broad details shown in Costs of melanoma section. |
| Sampling uncertainty | IN | NA – not a trial, will depend on specific evaluation |
| Subgroup analysis/Analysis of heterogeneity | IN | NA – not a trial, will depend on specific evaluation |
| Sensitivity analyses | IN | NA – not a trial, will depend on specific evaluation |
| Post hoc analyses | OUT | No. This manuscript is a protocol for model development and does not include specific cost-effectiveness or trial analyses. |
| Extrapolation or decision analytic modeling | IN | Yes. See Methods and analysis section. |
| Model type | IN | Yes. See Methods and analysis section. |
| Model structure | IN | Yes. See Figure 1. |
| Treatment effect beyond the end of the trial | IN | NA |
| Other key assumptions | IN | Yes. See Table 1. |
| Methods for identifying and estimating parameters | IN | Yes. See Methods and analysis (Data inputs) section. |
| Model uncertainty | IN | Yes. See Methods and analysis (Data inputs) section. |
| Model validation | IN | Yes. See Methods and analysis (Data inputs) section. |
| Subgroup analyses/Heterogeneity | IN | NA –will depend on specific evaluation |
| Value of information analysis | OPTIONAL LIST | No |
| Responsibility for health economic results and reporting | OUT | No |
| Reporting standards | IN | No. There are no results reported in this protocol paper. |
| Reporting deviations from the HEAP | IN | NA – not a trial |
| References to trial and statistical master file | OUT | NA – not a trial |
| References to other trial documents | OPTIONAL LIST | NA – not a trial |
| Appendices: Resource use data collected | IN | Table 1 and Costs of melanoma section |
| Appendices: Reporting checklists | OUT | No |
| Appendices: Illustrations | OPTIONAL LIST | Yes |

**Reviewer #2:**

The paper outlines a protocol for a microsimulation model intended to evaluate multiple potential screening strategies. The abstract mentions that the model aims to assess cost-effectiveness. However, I have several comments that I believe should be addressed before the manuscript can be considered for publication.

The abstract indicates that the model is to be used for cost-effectiveness assessments. It would be beneficial to include a clearer and more explicit statement of this purpose within the main text. More importantly, if the primary aim is to assess cost-effectiveness, there appear to be significant omissions in the provided information:

- Descriptions of cost data and quality of life data are absent. These are fundamental elements for cost-effectiveness analysis, and the authors must provide detailed information about them.
- According to guidelines for conducting cost-effectiveness analysis*, the authors should include additional details regarding:
  - The perspective of the model: Specify whether the analysis adopts a healthcare perspective or a societal perspective.
  - Handling uncertainty: Explain how the model addresses uncertainty related to both analytical modeling assumptions and the evidence used in the model.

See e.g.doi : /10.1016/j.jval.2012.06.012

Author response:

We thank Reviewer 2 for their comments. The protocol we have developed is for a generalisable modelling platform simulating melanoma, with the aim to apply this platform to relevant cost-effectiveness analyses upon its completion. We have edited the text in the Abstract and at the end of the Introduction to make this clearer.

The omissions that Reviewer 2 notes are with respect to the CHEERS checklist for cost-effectiveness analyses. As we have not completed a cost-effectiveness analysis within this protocol manuscript, we have not followed a CHEERS format. We have, however, now added a section containing information on the perspective of the model and costs and quality of life data that we intend to use in future cost-effectiveness analyses (see Costs of melanoma section, page 13). We have similarly added a section to the manuscript on how we propose to model the uncertainty of parameters in Policy1-Melanoma to address Reviewer 2’s concerns on the absence of information on handling modelling uncertainty (see ‘Data inputs’ section).
