# Supplementary material for: A protocol for development of a microsimulation model platform to evaluate the potential benefits, harms, and cost-effectiveness of risk-tailored melanoma screening
Source: PLoS One. 2025 Dec 26;20(12):e0339177. doi: 10.1371/journal.pone.0339177 (PMC12742747; doi:10.1371/journal.pone.0339177)
Supplement: Attachment — Submitted filename: Reaction_to_authors_R1.docx [file pone.0339177.s004.docx]

# Reactions to authors’ responses

I would like to thank the authors for taking the time to address my comments. However, I think some aspects of the text can still be improved to ensure the methods are clearly described. I first react on the authors’ reponses using the same numbering as the previous review, I have also some additional questions that arose when reading the manuscript again.

1. I think I still do not understand fully the method that the authors will use, and I think it has to do with the use of the term ‘transition probabilities’. I understand that the authors will use event-specific time-to-event distributions, and that the earliest event predicted will occur. But how will the time-to-event distributions be parameterized? What do the authors mean by ‘predicted based on transition probabilities’? Most often, the time-to-event distribution are parametric distributions such as exponential, Weibull, etc. distributions. Do the authors already have an idea of which type of distribution will be tested (on empirical/ published data?) and how the ‘best fitting’ distributions will be selected to determine the time to events?
   a. See the following papers concerning the different methods that can be used to address competing events: <https://doi.org/10.1177/0272989x18814770>, <https://doi.org/10.1016/j.jval.2021.07.016>, <https://doi.org/10.3389/fphar.2023.1255021>
2. Thank you for adding these details.
   a. The authors write: “*The probability of melanoma being diagnosed at a screening event depends upon the stage, histology, and location of the undiagnosed tumour”*. I guess that the sensitivity and specificity of the screening strategy will also be taken into account?
   b. Also, the authors write: *“The improved diagnostic rates from dermoscopy (or other diagnostic technologies) are also included as period effects in screening detection probability.”* 🡪 Does this mean that the sensitivity and specificity of dermoscopy have improved over time? And that this will be considered in the health economic model?
3. Thank you for adding the HEAP as a checklist, would it be possible to add it as supplementary material?

Additional remarks:

- Page 6, the authors write : “The modelling platform will be used to investigate many scenarios within different populations.”. I would suggest to remove this sentence since it is clear that the authors do not know yet which analyses will be performed with the to-be-developed model. This sentence does not add any clarity on this and is a bit vague.
- Table 1, assumptions “No skin checks to skin checks”, the authors write “Limited data available; we will estimate this through discussion with clinicians”. Would it even be valuable to perform a structured expert elicitation to estimate compliance to skin check?
- Table 1: the authors seem to be willing to use birth cohort before the 1980, but the authors also state that everybody in the model starts at the age 15 (birth cohort 2011 in 2026 when the model will be available). I do not understand why these old birth cohorts are needed in the current health economic model? Could the authors clarify?
- Page 9: I do not understand this sentence, could the authors clarify? “The impact of lifetime risk upon melanoma emergence is implemented as a scaling factor on age-dependent distributions of melanoma risk”
- Page 12; “The uncertainty associated with parameter selection will be **identified** through probabilistic sensitivity analyses and value of information analyses”. I suggest to replace “identified” by “assessed” since probabilistic analyses are used to show the impact of parameter uncertainty on the results, not only to identify it.
- As mentioned by the other reviewer in previous review round, there is no information on the quality of life weights that will be used in the model. Where will these come from?
- There is no detail provided on the methods and/ or tools that will be used to validate the model (e.g. Advishe, TECH-VER, PACBOARD). Also the description of the calibration methodology is succinct, please add a reference to the potential method(s) that will be used to calibrate model inputs.

Please incorporate a section on calibration and validation in the manuscript since both aspects are clearly mentioned as part of the aim of the protocol “Here we present a protocol for the development, calibration, and validation of a microsimulation platform to predict melanoma burden”

Typo’s:

- Page 4, Line 1: “charact eristics” -- > “characteristics”
- Page 4: bracket not closed “(2020/ETH03109; data accessed from 01/01/2022.”
- Page 4, correct punctuation? “melanomas; Fig 1)”
