# Supplementary material for: A protocol for development of a microsimulation model platform to evaluate the potential benefits, harms, and cost-effectiveness of risk-tailored melanoma screening
Source: PLoS One. 2025 Dec 26;20(12):e0339177. doi: 10.1371/journal.pone.0339177 (PMC12742747; doi:10.1371/journal.pone.0339177)
Supplement: Attachment — Submitted filename: ResponsesToReviewers_2025-05-24.docx [file pone.0339177.s006.docx]

# Reviewer 1

## Reactions to authors’ responses

**I would like to thank the authors for taking the time to address my comments. However, I think some aspects of the text can still be improved to ensure the methods are clearly described. I first react on the authors’ responses using the same numbering as the previous review, I have also some additional questions that arose when reading the manuscript again.**

We thank the reviewer for the helpful feedback and suggestions.

1. **I think I still do not understand fully the method that the authors will use, and I think it has to do with the use of the term ‘transition probabilities’. I understand that the authors will use event-specific time-to-event distributions, and that the earliest event predicted will occur. But how will the time-to-event distributions be parameterized? What do the authors mean by ‘predicted based on transition probabilities’? Most often, the time-to-event distribution are parametric distributions such as exponential, Weibull, etc. distributions. Do the authors already have an idea of which type of distribution will be tested (on empirical/ published data?) and how the ‘best fitting’ distributions will be selected to determine the time to events?**
   a. **See the following papers concerning the different methods that can be used to address competing events:** [**https://doi.org/10.1177/0272989x18814770**](https://doi.org/10.1177/0272989x18814770)**,** [**https://doi.org/10.1016/j.jval.2021.07.016**](https://doi.org/10.1016/j.jval.2021.07.016)**,** [**https://doi.org/10.3389/fphar.2023.1255021**](https://doi.org/10.3389/fphar.2023.1255021)

Thank you for your feedback requesting clarification on how transition probabilities can be used to calculate time-to-event distributions. Transition probabilities, which quantify the likelihood of moving between states over a specified time interval (e.g. one year), can serve as a foundation for calculating time-to-event distributions in probabilistic models. These distributions describe the probability of an event occurring by a specific time. By leveraging transition probabilities, we can compute the time-to-event distribution functions flexibly, accommodating both parametric and non-parametric forms, depending on the modelling assumptions and data characteristics.

We updated the text in the methods section (page 5/6) as follows:

“Competing events within Policy1-Melanoma will be dealt with using event-specific distributions; the time at which each competing event occurs is sampled from these distributions (calculated from transition probabilities listed in Table 1) and the earliest occurring event is selected.”

2. **Thank you for adding these details.**
   a. **The authors write: “*****The probability of melanoma being diagnosed at a screening event depends upon the stage, histology, and location of the undiagnosed tumour”*. I guess that the sensitivity and specificity of the screening strategy will also be taken into account?**

Yes, the sensitivity and specificity of any diagnostic procedures used in a screening event will be taken into account.

We have updated the text as follows (page 10):

“The probability of melanoma being diagnosed at a screening event depends upon several factors including the stage, histology, and location of the undiagnosed tumour (location is assigned using appropriate probability distributions derived from relevant epidemiological data), as well as the sensitivity and specificity of any diagnostic procedures involved.”

b. **Also, the authors write: *“The improved diagnostic rates from dermoscopy (or other diagnostic technologies) are also included as period effects in screening detection probability.”* 🡪 Does this mean that the sensitivity and specificity of dermoscopy have improved over time? And that this will be considered in the health economic model?**

Yes – the sensitivity and specificity of dermoscopy (or other diagnostic technologies) have improved over time due to technological advances and better training in their use. These changes will be taken into account when the model is used to model historical outcomes (as part of the calibration/validation process) but will be fixed for future evaluations (using the latest data to inform current sensitivity and specificity).

3. **Thank you for adding the HEAP as a checklist, would it be possible to add it as supplementary material?**

Yes, we have added the checklist to the supplementary material (Supplementary Table 1) and have referred to this on page 5.

**Additional remarks:**

- **Page 6, the authors write : “****The modelling platform will be used to investigate many scenarios within different populations.” I would suggest to remove this sentence since it is clear that the authors do not know yet which analyses will be performed with the to-be-developed model. This sentence does not add any clarity on this and is a bit vague.**

The sentence has been removed.

- **Table 1, assumptions “No skin checks to skin checks”, the authors write “Limited data available; we will estimate this through discussion with clinicians”. Would it even be valuable to perform a structured expert elicitation to estimate compliance to skin check?**

If feasible, we will conduct structured expert elicitation. We have added this to Table 1.

- **Table 1: the authors seem to be willing to use birth cohort before the 1980, but the authors also state that everybody in the model starts at the age 15 (birth cohort 2011 in 2026 when the model will be available). I do not understand why these old birth cohorts are needed in the current health economic model? Could the authors clarify?**

Now mentioned on page 11 (under ‘Data inputs’), the older cohorts will be used for:

- Calibrating and validating Policy1-Melanoma with historical data.
- Conducting multi-cohort analyses, such as assessing the impact of a screening program in Australia over the next 50 years.
- **Page 9: I do not understand this sentence, could the authors clarify? “The impact of lifetime risk upon melanoma emergence is implemented as a scaling factor on age-dependent distributions of melanoma risk”**

We have removed this sentence.

- **Page 12; “The uncertainty associated with parameter selection will be identified through probabilistic sensitivity analyses and value of information analyses”. I suggest to** **replace “identified” by “assessed” since probabilistic analyses are used to show the impact of parameter uncertainty on the results, not only to identify it.**

We have replaced “identified” by “assessed” as suggested (page 12).

- **As mentioned by the other reviewer in previous review round, there is no information on the quality of life weights that will be used in the model. Where will these come from?**

We will source quality of life weights for Policy1-Melanoma from the existing literature.

We have added the following text (page 13):

“Outcome measures will include the incremental cost effectiveness ratio (ICER) per life years saved and the ICER per quality-adjusted life year (QALY) gained, with quality of life weights sourced from existing literature.^75,76^”

- **There is no detail provided on the methods and/ or tools that will be used to validate the model (e.g. Advishe, TECH-VER, PACBOARD). Also the description of the calibration methodology is succinct, please add a reference to the potential method(s) that will be used to calibrate model inputs.**

**Please incorporate a section on calibration and validation in the manuscript since both aspects are clearly mentioned as part of the aim of the protocol “Here we present a protocol for the development, calibration, and validation of a microsimulation platform to predict melanoma burden.”**

We have added the following text on page 12 describing our calibration/validation approach in more detail:

“We will calibrate Policy1-Melanoma using a Bayesian synthesis approach.^70,71^ This methodology follows a systematic process to integrate multiple data sources to estimate model parameters while quantifying associated uncertainties. We will first establish prior distributions for model parameters informed by existing literature and clinician consultation. Subsequently, likelihood functions will be employed to align simulated outputs with observed melanoma data, facilitating parameter updates via Bayesian inference. This approach enables the synthesis of heterogeneous data and yields uncertainty estimates for model outputs, ensuring a rigorous calibration process. Model refinement will involve iterative diagnostic evaluations and sensitivity analyses ^70,71^ to produce a well-calibrated model that accurately represents melanoma epidemiology”.

**Typo’s:**

- **Page 4, Line 1: “charact eristics” -- > “characteristics”**
- **Page 4: bracket not closed “(2020/ETH03109; data accessed from 01/01/2022.”**
- **Page 4, correct punctuation? “melanomas; Fig 1)”**

All typos have been corrected.

# Reviewer 3

**This protocol paper outlines a microsimulation model for assessing the outcomes, costs, and cost-effectiveness of risk-stratified melanoma screening. Melanoma development and detection vary based on histological categories, as well as individual risk factors such as age, sex, and birth cohort. The paper underscores the global relevance and importance of evaluating melanoma screening programs and describes the model specifically within the Australian context.**

**The revisions to this paper have thoroughly addressed and incorporated the comments from Reviewers #1 and #2. The paper provides detailed information on the microsimulation model and the approach to evaluating the cost-effectiveness of melanoma screening.**

We thank the reviewer for the helpful feedback and suggestions.

**Two clarifications are worth considering for this manuscript:

Page 10: The probability of melanoma diagnosis is noted to depend on the location of the tumour (in addition to stage and histology). Further information on how tumour location will be incorporated into the model would be beneficial.**

To incorporate tumour location into the model, we will assign location using appropriate probability distributions derived from relevant epidemiological data, ensuring accurate representation of location-specific melanoma diagnosis probabilities alongside stage and histology.

We updated the text (page 10) as follows:

“The probability of melanoma being diagnosed at a screening event depends upon several factors including the stage, histology, and location of the undiagnosed tumour (location is assigned using appropriate probability distributions derived from relevant epidemiological data), as well as the sensitivity and specificity of any diagnostic procedures involved.”

**Page 12: Keratinocyte cancers and benign lesions are not included in the initial iteration of the model, but their diagnoses will be considered in the melanoma screening cost-effectiveness evaluation. Additional explanation would be helpful to clarify how these non-modelled components will be incorporated into the evaluation. It may be worth moving this paragraph earlier in the paper, as this information relates to the incidence and detection of skin cancers more generally.**

In the melanoma screening cost-effectiveness evaluations, keratinocyte cancers and benign lesions, although not explicitly included in Policy1-Melanoma, will be accounted for in the cost estimates. Specifically, for each simulated skin check, we will incorporate the associated costs of keratinocyte cancers and benign lesions based on their expected prevalence and treatment expenses. These costs will be integrated into the overall cost-effectiveness analysis to ensure a comprehensive evaluation.

We updated the text (page 13) as follows:

“Keratinocyte cancers and benign lesions will not be explicitly simulated in the initial iteration of the model. However, when evaluating the cost-effectiveness of risk-based melanoma screening programs, we will consider the financial implications of diagnosing these cancers and lesions in individuals participating in the program. Specifically, for each simulated skin check, we will incorporate the associated costs of keratinocyte cancers and benign lesions based on their expected prevalence and treatment expenses.^72^ Keratinocyte cancers and benign lesions will be incorporated into subsequent versions of the model.”
