# Supplementary material for: A protocol for development of a microsimulation model platform to evaluate the potential benefits, harms, and cost-effectiveness of risk-tailored melanoma screening
Source: PLoS One. 2025 Dec 26;20(12):e0339177. doi: 10.1371/journal.pone.0339177 (PMC12742747; doi:10.1371/journal.pone.0339177)
Supplement: Attachment — Submitted filename: ResponseToReviewers_20250807.docx [file pone.0339177.s007.docx]

Note – there were no comments from Reviewer 1 or Reviewer 2.

# Reviewer 3

**Thanks for addressing the remaining queries. I look forward to seeing this valuable protocol published.**

No changes required.

# Reviewer 4

**You are presenting a protocol for the development, calibration, and validation of a microsimulation platform to predict melanoma burden. Can this protocol be used by any country, other than Australia, given they have or can approximate the necessary inputs at the national level? You can respond by giving reference to Table 1, and perhaps better, include that information in Table 1 itself, since its title has “Australia” in it which suggests that the protocol and model assumptions , and particularly the model structure are more specific to Australia. I suppose the melanoma natural disease history models should be more generic, and distinction between countries may appear either in parameter values, or some policy/ scenario feasibilities.. etc, but less for structural elements of the model.**

We agree with the reviewer that melanoma natural history disease models should ideally be generalisable across contexts. However, the transition assumptions in Table 1 of our protocol are informed by Australian data. It is possible that some parameters will vary across countries, given different UV exposure, incidence and mortality rates, public health campaigns and sun-related behaviours. For the protocol to be applied in other countries, these assumptions must be validated using country-specific data. Thus, the protocol is transferable provided that relevant local data are available and the transition assumptions in Table 1, validated for Australia, are confirmed to be applicable.

We added the following to the Discussion section (page 15): "While the proposed protocol is tailored to the Australian context, its structure can be adapted for other countries by validating the transition assumptions in Table 1 and parameterising the model with country-specific data.”

**Your natural disease history model for melanoma: How does it compare with existing natural disease history models for melanoma? Can you provide any references to existing study/ protocol/ modeling papers related to melanoma modeling? I remember some as part of MCED modeling studies, as well as some single cancer screening papers. You can perhaps add mention these references by making reference to your main Figure 1.**

In a recently published systematic review (<https://doi.org/10.1186/s12911-025-03074-9>), we have looked at published microsimulation models of skin cancer and conducted a narrative synthesis of these models to assess their structures, parametrisation and assumptions. The structure we propose in this protocol paper addresses several limitations identified in our review.

We added the following sentence to the Methods and Analysis section (bottom of page 4): “This natural history structure addresses key limitations identified in a recent systematic review, which synthesised the structures, parameterisation, and assumptions of existing models.^18^”

**Your natural disease history and disease progression diagram is an important figure, can you increase resolution to make it more readable? And show potential “recovery” on the figure if it doesn’t get it very crowded? Now it feels like people don’t recover, and everybody gets diagnosed, there are also “undiagnosed” ones..**

We have now increased the resolution of the image. We appreciate the reviewer’s suggestion to explicitly depict “recovery” and “undiagnosed” cases in the natural history diagram to better reflect melanoma outcomes. In the current diagram (Figure 1), recovery is implicitly captured, as individuals who do not die from melanoma remain in earlier health states. Similarly, undiagnosed cases are incorporated within the histological states, as the model does not assume universal disease diagnosis. We believe these implicit representations, described in Figure 1’s caption, maintain the diagram’s clarity and simplicity while accurately reflecting the disease’s natural history. We considered adding ‘death from other causes’ to the figure, but this would need to be added in multiple places, making the figure very crowded and more difficult to read.

**Keywords: you may add a few mesh-terms instead of the existing ones which are not mesh-terms, e.g. “****early detection of cancer”, see for others: https://www.ncbi.nlm.nih.gov/mesh/**

We have now added MeSH terms as Keywords (page 2).

**Melanoma incidence: The global incidence is 2%, as we understand. You are stating that “This is particularly true in Australia and New Zealand, where despite incidence rates in young people stabilising, melanoma incidence remains the highest in the world”. Can you state the prevalence of the disease specifically, e.g. is it 4%? And perhaps same here, where you mention the incidence rates: “….which have been credited with reducing melanoma incidence in people under the age of 40 years”**

We have addressed this comment by adding more details to the text:

Page 3: “This is particularly true in Australia, where melanoma incidence remains the highest in the world^3^ and represents 11% of new invasive cancer diagnoses.^11^”

Page 6: “Since 1980, Australia has implemented many public health programs to reduce sun exposure, which have been credited with reducing melanoma incidence in people under the age of 40 years.^29,30^ Some of the reduction in incidence may also be due to changes in the ancestral composition of the Australian population.^31^ Nevertheless, despite the risk of being diagnosed with melanoma by the age of 30 in Australia halving since 1982, it remains the most common cancer among young adults aged 20-39 years and represents 16% of new invasive cancer diagnoses for that age-group.^11^

**Some of the text can be stated as a footnote, or moved to Appendix. E.g. this info can be a footnote. “…Tumours are classified using ICD-O-3 for body sites C44.0-C44.9 (and C80.9 melanoma of unknown primary site if not coded C44.9) and morphology codes M872-M879 with behaviour code 2 (in situ) or 3 (invasive).”**

We have moved this text to the Figure 1 description.

**Abbreviations: You have already defined USPSTF, the U.S. Preventive Services Task Force, on page 15, you do not need to spell it out in the text after that, try to stick to a standardized use, and for all abbreviations. It is spelled as U.S. and not US in text.**

We have removed the abbreviation USPSTF since it only appeared twice, and changed US to U.S.

**You can say “expert opinion “we will estimate this through discussion with clinicians and from cohort studies”.**

We have changed “discussion with clinicians” to “expert opinion”.

**Add a “List of Abbreviations” , since you have used many in text.**

Rather than provide a list of abbreviations, we have removed most abbreviations in the text to make it easier to read.

**We need to have more of these modeling papers/ protocols on cancer early detection, particularly for understudied types of cancers. I wish you all best with this study.**

Thank you!
