# Supplementary material for: A protocol for development of a microsimulation model platform to evaluate the potential benefits, harms, and cost-effectiveness of risk-tailored melanoma screening
Source: PLoS One. 2025 Dec 26;20(12):e0339177. doi: 10.1371/journal.pone.0339177 (PMC12742747; doi:10.1371/journal.pone.0339177)
Supplement: Peer review history

# Peer review: Decision Letter 0

**DOI:** 10.1371/journal.pone.0339177.r001

**Authors:** Wenpeng You

4 Dec 2024

Dear Dr. Cust,

plosone@plos.org . When you're ready to submit your revision, log on to https://www.editorialmanager.com/pone/ and select the 'Submissions Needing Revision' folder to locate your manuscript file.

- A rebuttal letter that responds to each point raised by the academic editor and reviewer(s). You should upload this letter as a separate file labeled 'Response to Reviewers'.
- A marked-up copy of your manuscript that highlights changes made to the original version. You should upload this as a separate file labeled 'Revised Manuscript with Track Changes'.
- An unmarked version of your revised paper without tracked changes. You should upload this as a separate file labeled 'Manuscript'.

If you would like to make changes to your financial disclosure, please include your updated statement in your cover letter. Guidelines for resubmitting your figure files are available below the reviewer comments at the end of this letter.

If applicable, we recommend that you deposit your laboratory protocols in protocols.io to enhance the reproducibility of your results. Protocols.io assigns your protocol its own identifier (DOI) so that it can be cited independently in the future. For instructions see: https://journals.plos.org/plosone/s/submission-guidelines#loc-laboratory-protocols . Additionally, PLOS ONE offers an option for publishing peer-reviewed Lab Protocol articles, which describe protocols hosted on protocols.io. Read more information on sharing protocols at https://plos.org/protocols?utm_medium=editorial-email&utm_source=authorletters&utm_campaign=protocols .

We look forward to receiving your revised manuscript.

Kind regards,

Wenpeng You

Academic Editor

PLOS ONE

Journal Requirements:

When submitting your revision, we need you to address these additional requirements.

1. Please ensure that your manuscript meets PLOS ONE's style requirements, including those for file naming. The PLOS ONE style templates can be found at

https://journals.plos.org/plosone/s/file?id=wjVg/PLOSOne_formatting_sample_main_body.pdf and

https://journals.plos.org/plosone/s/file?id=ba62/PLOSOne_formatting_sample_title_authors_affiliations.pdf

2. Thank you for stating the following financial disclosure:

"This project was supported by a National Health and Medical Research Council (NHMRC) project grant (#1165936 to CGW, PHS, PF, DCW, PG, KC, AEC, MC) and NHMRC Synergy grant (#2009923 to PHS, PF, KC, AEC). AEC is supported by a NHMRC Investigator Grant (#2008454). www.nhmrc.gov.au/"

Please state what role the funders took in the study.  If the funders had no role, please state: ""The funders had no role in study design, data collection and analysis, decision to publish, or preparation of the manuscript.""

If this statement is not correct you must amend it as needed.

Please include this amended Role of Funder statement in your cover letter; we will change the online submission form on your behalf.

[Note: HTML markup is below. Please do not edit.]

Reviewers' comments:

Reviewer's Responses to Questions

**Comments to the Author**

1. Does the manuscript provide a valid rationale for the proposed study, with clearly identified and justified research questions?

Reviewer #1: Yes

Reviewer #2: Yes

**********

2. Is the protocol technically sound and planned in a manner that will lead to a meaningful outcome and allow testing the stated hypotheses?

Reviewer #1: Partly

Reviewer #2: Yes

**********

3. Is the methodology feasible and described in sufficient detail to allow the work to be replicable?

Reviewer #1: Yes

Reviewer #2: No

**********

4. Have the authors described where all data underlying the findings will be made available when the study is complete?

The PLOS Data policy

Reviewer #1: Yes

Reviewer #2: No

**********

5. Is the manuscript presented in an intelligible fashion and written in standard English?

Reviewer #1: Yes

Reviewer #2: Yes

**********

Please use the space provided to explain your answers to the questions above and, if applicable, provide comments about issues authors must address before this protocol can be accepted for publication. You may also include additional comments for the author, including concerns about research or publication ethics.

You may also provide optional suggestions and comments to authors that they might find helpful in planning their study.

Reviewer #1: Dear all,

Thank you for providing me with the opportunity of reviewing this nice protocol paper for the development of a health economic model. I would really like to congratulate the authors on taking up this initiative since I think we, as a field, take too little time to write such protocols for the development of such models, while they are a valuable tool to think about structural assumptions of a model and potentially prevent duplication of work. I think this type of initiatives should be praised within the health economics and outcomes research field.

Please find in the attached PDF file my detailed comments on the text.

My main comments are the following:

1. The authors mention in their manuscript that they will develop a discrete event simulation. However they do not provide any information on which time-to-event distributions will be considered and how competing events will be addressed within the health economic model. Please add a section on these issues.

2. The authors describe certain aspects of melanoma screening in quite general terms and do not always relate this information to how it will be included in the health economic model, please be more specific on how the information mentioned in the manuscript informs structural assumptions or inputs of the health economic model.

3. I would like to suggest to structure the text of this protocol according to the “health economics analysis plan” described in Thorn et al. since I think not all relevant aspects of the model are equally thoroughly mentioned (Thorn JC, Davies CF, Brookes ST, Noble SM, Dritsaki M, Gray E, Hughes DA, Mihaylova B, Petrou S, Ridyard C, Sach T. Content of Health Economics Analysis Plans (HEAPs) for trial-based economic evaluations: expert Delphi consensus survey. Value in Health. 2021 Apr 1;24(4):539-47.). Even though this reference seems to focus on trial-based economic evaluations, it also contains a section dedicated to health economic models, and of course the general sections apply to this protocol as well.

Good luck with updating this manuscript!

Xavier Pouwels

Reviewer #2: The paper outlines a protocol for a microsimulation model intended to evaluate multiple potential screening strategies. The abstract mentions that the model aims to assess cost-effectiveness. However, I have several comments that I believe should be addressed before the manuscript can be considered for publication.

The abstract indicates that the model is to be used for cost-effectiveness assessments. It would be beneficial to include a clearer and more explicit statement of this purpose within the main text. More importantly, if the primary aim is to assess cost-effectiveness, there appear to be significant omissions in the provided information:

• Descriptions of cost data and quality of life data are absent. These are fundamental elements for cost-effectiveness analysis, and the authors must provide detailed information about them.

• According to guidelines for conducting cost-effectiveness analysis*, the authors should include additional details regarding:

o The perspective of the model: Specify whether the analysis adopts a healthcare perspective or a societal perspective.

o Handling uncertainty: Explain how the model addresses uncertainty related to both analytical modeling assumptions and the evidence used in the model.

See e.g.doi : /10.1016/j.jval.2012.06.012

**********

what does this mean? ). If published, this will include your full peer review and any attached files.

If you choose “no”, your identity will remain anonymous but your review may still be made public.

**Do you want your identity to be public for this peer review?** For information about this choice, including consent withdrawal, please see our Privacy Policy

Reviewer #1: **Yes: ** Xavier Pouwels

Reviewer #2: No

**********

[NOTE: If reviewer comments were submitted as an attachment file, they will be attached to this email and accessible via the submission site. Please log into your account, locate the manuscript record, and check for the action link "View Attachments". If this link does not appear, there are no attachment files.]

While revising your submission, please upload your figure files to the Preflight Analysis and Conversion Engine (PACE) digital diagnostic tool, https://pacev2.apexcovantage.com/ . PACE helps ensure that figures meet PLOS requirements. To use PACE, you must first register as a user. Registration is free. Then, login and navigate to the UPLOAD tab, where you will find detailed instructions on how to use the tool. If you encounter any issues or have any questions when using PACE, please email PLOS at figures@plos.org . Please note that Supporting Information files do not need this step.

Attachment Submitted filename: PONE-D-24-40002_reviewer.pdf

# Author comment: Author response to Decision Letter 1

**DOI:** 10.1371/journal.pone.0339177.r002

22 Jan 2025

*********(see also attached Response to reviewers document)****************

Journal Requirements:

1. Please ensure that your manuscript meets PLOS ONE's style requirements, including those for file naming.

Author response: We comply with the PLOS ONE style requirements.

2. Thank you for stating the following financial disclosure:

"This project was supported by a National Health and Medical Research Council (NHMRC) project grant (#1165936 to CGW, PHS, PF, DCW, PG, KC, AEC, MC) and NHMRC Synergy grant (#2009923 to PHS, PF, KC, AEC). AEC is supported by a NHMRC Investigator Grant (#2008454). www.nhmrc.gov.au/"

Author response: We have added this financial disclosure on page 14 immediately before the References.

Please state what role the funders took in the study. If the funders had no role, please state: ""The funders had no role in study design, data collection and analysis, decision to publish, or preparation of the manuscript.""

If this statement is not correct you must amend it as needed.

Please include this amended Role of Funder statement in your cover letter; we will change the online submission form on your behalf.

Author response: We have added this sentence to the financial disclosure on page 14 and to the cover letter.

Reviewers' comments:

Reviewer's Responses to Questions

Comments to the Author

1. Does the manuscript provide a valid rationale for the proposed study, with clearly identified and justified research questions?

The research question outlined is expected to address a valid academic problem or topic and contribute to the base of knowledge in the field.

Reviewer #1: Yes

Reviewer #2: Yes

________________________________________

2. Is the protocol technically sound and planned in a manner that will lead to a meaningful outcome and allow testing the stated hypotheses?

The manuscript should describe the methods in sufficient detail to prevent undisclosed flexibility in the experimental procedure or analysis pipeline, including sufficient outcome-neutral conditions (e.g. necessary controls, absence of floor or ceiling effects) to test the proposed hypotheses and a statistical power analysis where applicable. As there may be aspects of the methodology and analysis which can only be refined once the work is undertaken, authors should outline potential assumptions and explicitly describe what aspects of the proposed analyses, if any, are exploratory.

Reviewer #1: Partly

Reviewer #2: Yes

________________________________________

3. Is the methodology feasible and described in sufficient detail to allow the work to be replicable?

Descriptions of methods and materials in the protocol should be reported in sufficient detail for another researcher to reproduce all experiments and analyses. The protocol should describe the appropriate controls, sample size calculations, and replication needed to ensure that the data are robust and reproducible.

Reviewer #1: Yes

Reviewer #2: No

________________________________________

4. Have the authors described where all data underlying the findings will be made available when the study is complete?

The PLOS Data policy requires authors to make all data underlying the findings described in their manuscript fully available without restriction, with rare exception, at the time of publication. The data should be provided as part of the manuscript or its supporting information, or deposited to a public repository. For example, in addition to summary statistics, the data points behind means, medians and variance measures should be available. If there are restrictions on publicly sharing data—e.g. participant privacy or use of data from a third party—those must be specified.

Reviewer #1: Yes

Reviewer #2: No

________________________________________

5. Is the manuscript presented in an intelligible fashion and written in standard English?

PLOS ONE does not copyedit accepted manuscripts, so the language in submitted articles must be clear, correct, and unambiguous. Any typographical or grammatical errors should be corrected at revision, so please note any specific errors here.

Reviewer #1: Yes

Reviewer #2: Yes

________________________________________

6. Review Comments to the Author

Please use the space provided to explain your answers to the questions above and, if applicable, provide comments about issues authors must address before this protocol can be accepted for publication. You may also include additional comments for the author, including concerns about research or publication ethics.

You may also provide optional suggestions and comments to authors that they might find helpful in planning their study.

(Please upload your review as an attachment if it exceeds 20,000 characters)

Reviewer #1:

Dear all,

Thank you for providing me with the opportunity of reviewing this nice protocol paper for the development of a health economic model. I would really like to congratulate the authors on taking up this initiative since I think we, as a field, take too little time to write such protocols for the development of such models, while they are a valuable tool to think about structural assumptions of a model and potentially prevent duplication of work. I think this type of initiatives should be praised within the health economics and outcomes research field.

Please find in the attached PDF file my detailed comments on the text.

Author response: Thank you Prof. Pouwels for attaching your detailed comments marked on the manuscript file. We have gone through each comment and made changes in the text in response. We have also responded to your specific comments below.

My main comments are the following:

1. The authors mention in their manuscript that they will develop a discrete event simulation. However they do not provide any information on which time-to-event distributions will be considered and how competing events will be addressed within the health economic model. Please add a section on these issues.

Author response:

We have added the following details to the text (page 5): “Competing events within Policy1-Melanoma will be dealt with using event-specific distributions; the time at which each competing event occurs is predicted based on transition probabilities and the earliest occurring event is selected.22”

2. The authors describe certain aspects of melanoma screening in quite general terms and do not always relate this information to how it will be included in the health economic model, please be more specific on how the information mentioned in the manuscript informs structural assumptions or inputs of the health economic model.

Author response:

We have made edits in the manuscript to address this, for example page 9-10 where we describe how the effect of changing diagnostic technologies is incorporated into the model: “The effect of changing diagnostic techniques is incorporated into Policy1-Melanoma as a scaling function, improving diagnostic rates until they match observed diagnostic technology usage in Australia. Screening within Policy1-Melanoma is implemented as a detection event. Individuals ‘attend’ a screening event at which, if they have an undiagnosed melanoma, melanoma can be diagnosed. The probability of melanoma being diagnosed at a screening event depends upon the stage, histology, and location of the undiagnosed tumour. The improved diagnostic rates from dermoscopy (or other diagnostic technologies) are also included as period effects in screening detection probability.”

We note that the modelling platform will be able to be used to investigate many scenarios within different populations. We hope that by designing a simulation which is more generalisable than standard health economic models we will be able to use the platform to answer policy questions which have not yet arisen.

3. I would like to suggest to structure the text of this protocol according to the “health economics analysis plan” described in Thorn et al. since I think not all relevant aspects of the model are equally thoroughly mentioned (Thorn JC, Davies CF, Brookes ST, Noble SM, Dritsaki M, Gray E, Hughes DA, Mihaylova B, Petrou S, Ridyard C, Sach T. Content of Health Economics Analysis Plans (HEAPs) for trial-based economic evaluations: expert Delphi consensus survey. Value in Health. 2021 Apr 1;24(4):539-47.). Even though this reference seems to focus on trial-based economic evaluations, it also contains a section dedicated to health economic models, and of course the general sections apply to this protocol as well. Good luck with updating this manuscript! Xavier Pouwels

Author response:

Thank you for this suggestion. We agree that it is important that models are reported clearly, and we have made changes to the manuscript throughout to improve its clarity. For example, we have now included a new section included on ‘Costs of melanoma’ (page 13 before the Discussion), and more details in the ‘Data inputs’ section. We show the proposed checklist from Thorn et al (2021) below, and indicate where the relevant information is contained within the manuscript. Where information was missing, this has now been added to the manuscript. If an included component is missing from the manuscript we have indicated why it has not been incorporated or is not relevant. Whilst we have incorporated additional components into the text, our preference is to retain our current manuscript structure, as this enables us to better highlight the conceptual uniqueness of melanoma simulations (e.g. as a visible, high-survival, potentially repeatable cancer) when compared to simulations of other cancer types.

Thorn et al (2021) expected content of health economic analysis plans.

Item Item status after final voting Item included in protocol?

Title IN Yes.

Trial registration number IN NA – not a trial

Source of funding IN Yes - added

Purpose of health economics analysis plan (HEAP) IN NA –not a trial, but the Aims of the protocol are specified in the Abstract and Introduction

Sponsor approval OUT No

Trial protocol version IN NA – not a trial

Trial statistical analysis plan (SAP) version IN NA – not a trial

Trial HEAP version IN NA – not a trial

HEAP revisions IN NA – not a trial

Table of contents OPTIONAL LIST No

Abbreviations/glossary of terms/definitions OPTIONAL LIST No

Roles and responsibilities IN Yes. See Authors Contributions section.

Signature(s) of person(s) writing HEAP (and date) IN NA – not a trial

Signature of senior health economist (HE) who is guarantor of the economic evaluation (and date) IN NA – not a trial

Signature of the chief investigator for the trial IN NA – not a trial

Trial background and rationale IN NA. Background information in Introduction.

Aim(s) of the trial IN NA – this is not a trial, but the Aims of the protocol are specified in the Abstract and Introduction

Objectives and/or research hypotheses of the trial IN NA as above

Trial population IN Yes. See Introduction & Methods and analysis.

Intervention and comparator(s) IN NA. Protocol is for a simulation which can be used in multiple contexts.

Trial design IN NA – not a trial

Trial start and end dates IN NA – not a trial

Aim(s) of economic evaluation IN This is a protocol for a generalisable microsimulation model of melanoma that can be flexibly used to evaluate a range of scenarios related to melanoma screening, diagnosis, surveillance and management (rather than a specific economic evaluation). However, in the Abstract and Introduction we mention our specific aim to evaluate skin cancer screening.

Objectives(s)/hypotheses of economic evaluation IN As above

Overview of economic analysis IN Methods and Analysis

Jurisdiction IN Yes. See Introduction & Methods and analysis.

Perspective(s) IN Yes – see Costs of melanoma section.

Time horizon IN Yes – added lifetime time horizon. See Methods and analysis section – Natural history structure.

Monitoring collection of health economic data OPTIONAL LIST No.

Database management OPTIONAL LIST NA.

Data entry OPTIONAL LIST NA

Data cleaning for analysis IN No. Data was cleaned by the Australian Cancer Registries. Additional cleaning may be performed before economic analyses are performed and this information would be provided in the context of a published model and specific evaluations.

Data archiving OPTIONAL LIST NA

Statistical software used for HE analysis IN Yes - added. See Methods and analysis section.

Identification of resources IN Yes – see Costs of melanoma section.

Measurement of resource use data IN Yes - as above

Valuation of resource use data IN Yes - see Costs of melanoma section.

Identification of outcome(s) IN Yes - see Costs of melanoma section.

Measurement of outcome(s) IN Yes - see Costs of melanoma section. More details will be provided in later manuscripts showing results of specific evaluations.

Valuation of outcome(s) IN Yes - see Costs of melanoma section. More details will be provided in later manuscripts showing results of specific evaluations.

Analysis population IN See Methods and Analysis for broad population details

Timing of analyses IN No – as the analyses will be ongoing

Discount rates for costs and benefits IN Yes - see Costs of melanoma section.

Cost-effectiveness threshold(s) IN Yes - see Costs of melanoma section.

Statistical decision rule(s) IN NA – not a trial, will depend on specific evaluation

Analysis of resource use IN NA – not a trial, will depend on specific evaluation

Analysis of costs IN NA – not a trial, will depend on specific evaluation

Analysis of outcomes IN NA – not a trial, will depend on specific evaluation

Missing data IN NA – not a trial, will depend on specific evaluation

Analysis of cost-effectiveness IN Yes – broad details shown in Costs of melanoma section.

Sampling uncertainty IN NA – not a trial, will depend on specific evaluation

Subgroup analysis/Analysis of heterogeneity IN NA – not a trial, will depend on specific evaluation

Sensitivity analyses IN NA – not a trial, will depend on specific evaluation

Post hoc analyses OUT No. This manuscript is a protocol for model development and does not include specific cost-effectiveness or trial analyses.

Extrapolation or decision analytic modeling IN Yes. See Methods and analysis section.

Model type IN Yes. See Methods and analysis section.

Model structure IN Yes. See Figure 1.

Treatment effect beyond the end of the trial IN NA

Other key assumptions IN Yes. See Table 1.

Methods for identifying and estimating parameters IN Yes. See Methods and analysis (Data inputs) section.

Model uncertainty IN Yes. See Methods and analysis (Data inputs) section.

Model validation IN Yes. See Methods and analysis (Data inputs) section.

Subgroup analyses/Heterogeneity IN NA –will depend on specific evaluation

Value of information analysis OPTIONAL LIST No

Responsibility for health economic results and reporting OUT No

Reporting standards IN No. There are no results reported in this protocol paper.

Reporting deviations from the HEAP IN NA – not a trial

References to trial and statistical master file OUT NA – not a trial

References to other trial documents OPTIONAL LIST NA – not a trial

Appendices: Resource use data collected IN Table 1 and Costs of melanoma section

Appendices: Reporting checklists OUT No

Appendices: Illustrations OPTIONAL LIST Yes

Reviewer #2:

The paper outlines a protocol for a microsimulation model intended to evaluate multiple potential screening strategies. The abstract mentions that the model aims to assess cost-effectiveness. However, I have several comments that I believe should be addressed before the manuscript can be considered for publication.

The abstract indicates that the model is to be used for cost-effectiveness assessments. It would be beneficial to include a clearer and more explicit statement of this purpose within the main text. More importantly, if the primary aim is to assess cost-effectiveness, there appear to be significant omissions in the provided information:

• Descriptions of cost data and quality of life data are absent. These are fundamental elements for cost-effectiveness analysis, and the authors must provide detailed information about them.

• According to guidelines for conducting cost-effectiveness analysis*, the authors should include

Attachment Submitted filename: Responses_to_reviewers_20250122.docx

# Peer review: Decision Letter 1

**DOI:** 10.1371/journal.pone.0339177.r003

**Authors:** Wenpeng You

10 Apr 2025

Dear Dr. Cust,

plosone@plos.org . When you're ready to submit your revision, log on to https://www.editorialmanager.com/pone/ and select the 'Submissions Needing Revision' folder to locate your manuscript file.

- A rebuttal letter that responds to each point raised by the academic editor and reviewer(s). You should upload this letter as a separate file labeled 'Response to Reviewers'.
- A marked-up copy of your manuscript that highlights changes made to the original version. You should upload this as a separate file labeled 'Revised Manuscript with Track Changes'.
- An unmarked version of your revised paper without tracked changes. You should upload this as a separate file labeled 'Manuscript'.

If applicable, we recommend that you deposit your laboratory protocols in protocols.io to enhance the reproducibility of your results. Protocols.io assigns your protocol its own identifier (DOI) so that it can be cited independently in the future. For instructions see: https://journals.plos.org/plosone/s/submission-guidelines#loc-laboratory-protocols . Additionally, PLOS ONE offers an option for publishing peer-reviewed Lab Protocol articles, which describe protocols hosted on protocols.io. Read more information on sharing protocols at https://plos.org/protocols?utm_medium=editorial-email&utm_source=authorletters&utm_campaign=protocols .

We look forward to receiving your revised manuscript.

Kind regards,

Wenpeng You

Academic Editor

PLOS ONE

**Journal Requirements:**

Please review your reference list to ensure that it is complete and correct. If you have cited papers that have been retracted, please include the rationale for doing so in the manuscript text, or remove these references and replace them with relevant current references. Any changes to the reference list should be mentioned in the rebuttal letter that accompanies your revised manuscript. If you need to cite a retracted article, indicate the article’s retracted status in the References list and also include a citation and full reference for the retraction notice.

[Note: HTML markup is below. Please do not edit.]

Reviewers' comments:

Reviewer's Responses to Questions

**Comments to the Author**

1. Does the manuscript provide a valid rationale for the proposed study, with clearly identified and justified research questions?

Reviewer #1: Yes

Reviewer #3: Yes

**********

2. Is the protocol technically sound and planned in a manner that will lead to a meaningful outcome and allow testing the stated hypotheses?

Reviewer #1: Yes

Reviewer #3: Yes

**********

3. Is the methodology feasible and described in sufficient detail to allow the work to be replicable?

Reviewer #1: No

Reviewer #3: Yes

**********

4. Have the authors described where all data underlying the findings will be made available when the study is complete?

The PLOS Data policy

Reviewer #1: No

Reviewer #3: No

**********

5. Is the manuscript presented in an intelligible fashion and written in standard English?

Reviewer #1: Yes

Reviewer #3: Yes

**********

Please use the space provided to explain your answers to the questions above and, if applicable, provide comments about issues authors must address before this protocol can be accepted for publication. You may also include additional comments for the author, including concerns about research or publication ethics.

You may also provide optional suggestions and comments to authors that they might find helpful in planning their study.

**Reviewer #1:**  I would like to thank the authors for taking the time to address my comments. However, I think some aspects of the text can still be improved to ensure the methods are clearly described. I first react on the authors’ reponses using the same numbering as the previous review, I have also some additional questions that arose when reading the manuscript again.

1. I think I still do not understand fully the method that the authors will use, and I think it has to do with the use of the term ‘transition probabilities’. I understand that the authors will use event-specific time-to-event distributions, and that the earliest event predicted will occur. But how will the time-to-event distributions be parameterized? What do the authors mean by ‘predicted based on transition probabilities’? Most often, the time-to-event distribution are parametric distributions such as exponential, Weibull, etc. distributions. Do the authors already have an idea of which type of distribution will be tested (on empirical/ published data?) and how the ‘best fitting’ distributions will be selected to determine the time to events?

a. See the following papers concerning the different methods that can be used to address competing events: https://doi.org/10.1177/0272989x18814770, https://doi.org/10.1016/j.jval.2021.07.016, https://doi.org/10.3389/fphar.2023.1255021

2. Thank you for adding these details.

a. The authors write: “The probability of melanoma being diagnosed at a screening event depends upon the stage, histology, and location of the undiagnosed tumour”. I guess that the sensitivity and specificity of the screening strategy will also be taken into account?

b. Also, the authors write: “The improved diagnostic rates from dermoscopy (or other diagnostic technologies) are also included as period effects in screening detection probability.” � Does this mean that the sensitivity and specificity of dermoscopy have improved over time? And that this will be considered in the health economic model?

3. Thank you for adding the HEAP as a checklist, would it be possible to add it as supplementary material?

Additional remarks:

- Page 6, the authors write : “The modelling platform will be used to investigate many scenarios within different populations.”. I would suggest to remove this sentence since it is clear that the authors do not know yet which analyses will be performed with the to-be-developed model. This sentence does not add any clarity on this and is a bit vague.

- Table 1, assumptions “No skin checks to skin checks”, the authors write “Limited data available; we will estimate this through discussion with clinicians”. Would it even be valuable to perform a structured expert elicitation to estimate compliance to skin check?

- Table 1: the authors seem to be willing to use birth cohort before the 1980, but the authors also state that everybody in the model starts at the age 15 (birth cohort 2011 in 2026 when the model will be available). I do not understand why these old birth cohorts are needed in the current health economic model? Could the authors clarify?

- Page 9: I do not understand this sentence, could the authors clarify? “The impact of lifetime risk upon melanoma emergence is implemented as a scaling factor on age-dependent distributions of melanoma risk”

- Page 12; “The uncertainty associated with parameter selection will be identified through probabilistic sensitivity analyses and value of information analyses”. I suggest to replace “identified” by “assessed” since probabilistic analyses are used to show the impact of parameter uncertainty on the results, not only to identify it.

- As mentioned by the other reviewer in previous review round, there is no information on the quality of life weights that will be used in the model. Where will these come from?

- There is no detail provided on the methods and/ or tools that will be used to validate the model (e.g. Advishe, TECH-VER, PACBOARD). Also the description of the calibration methodology is succinct, please add a reference to the potential method(s) that will be used to calibrate model inputs.

Please incorporate a section on calibration and validation in the manuscript since both aspects are clearly mentioned as part of the aim of the protocol “Here we present a protocol for the development, calibration, and validation of a microsimulation platform to predict melanoma burden”

Typo’s:

- Page 4, Line 1: “charact eristics” -- > “characteristics”

- Page 4: bracket not closed? “(2020/ETH03109; data accessed from 01/01/2022.”

- Page 4, correct punctuation? “melanomas; Fig 1)”

Xavier Pouwels

**Reviewer #3:**  This protocol paper outlines a microsimulation model for assessing the outcomes, costs, and cost-effectiveness of risk-stratified melanoma screening. Melanoma development and detection vary based on histological categories, as well as individual risk factors such as age, sex, and birth cohort. The paper underscores the global relevance and importance of evaluating melanoma screening programs and describes the model specifically within the Australian context.

The revisions to this paper have thoroughly addressed and incorporated the comments from Reviewers #1 and #2. The paper provides detailed information on the microsimulation model and the approach to evaluating the cost-effectiveness of melanoma screening.

Two clarifications are worth considering for this manuscript:

Page 10: The probability of melanoma diagnosis is noted to depend on the location of the tumour (in addition to stage and histology). Further information on how tumour location will be incorporated into the model would be beneficial.

Page 12: Keratinocyte cancers and benign lesions are not included in the initial iteration of the model, but their diagnoses will be considered in the melanoma screening cost-effectiveness evaluation. Additional explanation would be helpful to clarify how these non-modelled components will be incorporated into the evaluation. It may be worth moving this paragraph earlier in the paper, as this information relates to the incidence and detection of skin cancers more generally.

**********

what does this mean? ). If published, this will include your full peer review and any attached files.

If you choose “no”, your identity will remain anonymous but your review may still be made public.

**Do you want your identity to be public for this peer review?** For information about this choice, including consent withdrawal, please see our Privacy Policy

Reviewer #1: **Yes: ** Xavier G.L.V. Pouwels

Reviewer #3: No

**********

[NOTE: If reviewer comments were submitted as an attachment file, they will be attached to this email and accessible via the submission site. Please log into your account, locate the manuscript record, and check for the action link "View Attachments". If this link does not appear, there are no attachment files.]

While revising your submission, please upload your figure files to the Preflight Analysis and Conversion Engine (PACE) digital diagnostic tool, https://pacev2.apexcovantage.com/ . PACE helps ensure that figures meet PLOS requirements. To use PACE, you must first register as a user. Registration is free. Then, login and navigate to the UPLOAD tab, where you will find detailed instructions on how to use the tool. If you encounter any issues or have any questions when using PACE, please email PLOS at figures@plos.org

Attachment Submitted filename: Reaction_to_authors_R1.docx

# Author comment: Author response to Decision Letter 2

**DOI:** 10.1371/journal.pone.0339177.r004

24 May 2025

This response to Reviewers' comments has also been uploaded as a separate document.

Reviewer 1

Reactions to authors’ responses

I would like to thank the authors for taking the time to address my comments. However, I think some aspects of the text can still be improved to ensure the methods are clearly described. I first react on the authors’ responses using the same numbering as the previous review, I have also some additional questions that arose when reading the manuscript again.

We thank the reviewer for the helpful feedback and suggestions.

1. I think I still do not understand fully the method that the authors will use, and I think it has to do with the use of the term ‘transition probabilities’. I understand that the authors will use event-specific time-to-event distributions, and that the earliest event predicted will occur. But how will the time-to-event distributions be parameterized? What do the authors mean by ‘predicted based on transition probabilities’? Most often, the time-to-event distribution are parametric distributions such as exponential, Weibull, etc. distributions. Do the authors already have an idea of which type of distribution will be tested (on empirical/ published data?) and how the ‘best fitting’ distributions will be selected to determine the time to events?

a. See the following papers concerning the different methods that can be used to address competing events: https://doi.org/10.1177/0272989x18814770, https://doi.org/10.1016/j.jval.2021.07.016, https://doi.org/10.3389/fphar.2023.1255021

Thank you for your feedback requesting clarification on how transition probabilities can be used to calculate time-to-event distributions. Transition probabilities, which quantify the likelihood of moving between states over a specified time interval (e.g. one year), can serve as a foundation for calculating time-to-event distributions in probabilistic models. These distributions describe the probability of an event occurring by a specific time. By leveraging transition probabilities, we can compute the time-to-event distribution functions flexibly, accommodating both parametric and non-parametric forms, depending on the modelling assumptions and data characteristics.

We updated the text in the methods section (page 5/6) as follows:

“Competing events within Policy1-Melanoma will be dealt with using event-specific distributions; the time at which each competing event occurs is sampled from these distributions (calculated from transition probabilities listed in Table 1) and the earliest occurring event is selected.”

2. Thank you for adding these details.

a. The authors write: “The probability of melanoma being diagnosed at a screening event depends upon the stage, histology, and location of the undiagnosed tumour”. I guess that the sensitivity and specificity of the screening strategy will also be taken into account?

Yes, the sensitivity and specificity of any diagnostic procedures used in a screening event will be taken into account.

We have updated the text as follows (page 10):

“The probability of melanoma being diagnosed at a screening event depends upon several factors including the stage, histology, and location of the undiagnosed tumour (location is assigned using appropriate probability distributions derived from relevant epidemiological data), as well as the sensitivity and specificity of any diagnostic procedures involved.”

b. Also, the authors write: “The improved diagnostic rates from dermoscopy (or other diagnostic technologies) are also included as period effects in screening detection probability.” � Does this mean that the sensitivity and specificity of dermoscopy have improved over time? And that this will be considered in the health economic model?

Yes – the sensitivity and specificity of dermoscopy (or other diagnostic technologies) have improved over time due to technological advances and better training in their use. These changes will be taken into account when the model is used to model historical outcomes (as part of the calibration/validation process) but will be fixed for future evaluations (using the latest data to inform current sensitivity and specificity).

3. Thank you for adding the HEAP as a checklist, would it be possible to add it as supplementary material?

Yes, we have added the checklist to the supplementary material (Supplementary Table 1) and have referred to this on page 5.

Additional remarks:

- Page 6, the authors write : “The modelling platform will be used to investigate many scenarios within different populations.” I would suggest to remove this sentence since it is clear that the authors do not know yet which analyses will be performed with the to-be-developed model. This sentence does not add any clarity on this and is a bit vague.

The sentence has been removed.

- Table 1, assumptions “No skin checks to skin checks”, the authors write “Limited data available; we will estimate this through discussion with clinicians”. Would it even be valuable to perform a structured expert elicitation to estimate compliance to skin check?

If feasible, we will conduct structured expert elicitation. We have added this to Table 1.

- Table 1: the authors seem to be willing to use birth cohort before the 1980, but the authors also state that everybody in the model starts at the age 15 (birth cohort 2011 in 2026 when the model will be available). I do not understand why these old birth cohorts are needed in the current health economic model? Could the authors clarify?

Now mentioned on page 11 (under ‘Data inputs’), the older cohorts will be used for:

• Calibrating and validating Policy1-Melanoma with historical data.

• Conducting multi-cohort analyses, such as assessing the impact of a screening program in Australia over the next 50 years.

- Page 9: I do not understand this sentence, could the authors clarify? “The impact of lifetime risk upon melanoma emergence is implemented as a scaling factor on age-dependent distributions of melanoma risk”

We have removed this sentence.

- Page 12; “The uncertainty associated with parameter selection will be identified through probabilistic sensitivity analyses and value of information analyses”. I suggest to replace “identified” by “assessed” since probabilistic analyses are used to show the impact of parameter uncertainty on the results, not only to identify it.

We have replaced “identified” by “assessed” as suggested (page 12).

- As mentioned by the other reviewer in previous review round, there is no information on the quality of life weights that will be used in the model. Where will these come from?

We will source quality of life weights for Policy1-Melanoma from the existing literature.

We have added the following text (page 13):

“Outcome measures will include the incremental cost effectiveness ratio (ICER) per life years saved and the ICER per quality-adjusted life year (QALY) gained, with quality of life weights sourced from existing literature.75,76”

- There is no detail provided on the methods and/ or tools that will be used to validate the model (e.g. Advishe, TECH-VER, PACBOARD). Also the description of the calibration methodology is succinct, please add a reference to the potential method(s) that will be used to calibrate model inputs.

Please incorporate a section on calibration and validation in the manuscript since both aspects are clearly mentioned as part of the aim of the protocol “Here we present a protocol for the development, calibration, and validation of a microsimulation platform to predict melanoma burden.”

We have added the following text on page 12 describing our calibration/validation approach in more detail:

“We will calibrate Policy1-Melanoma using a Bayesian synthesis approach.70,71 This methodology follows a systematic process to integrate multiple data sources to estimate model parameters while quantifying associated uncertainties. We will first establish prior distributions for model parameters informed by existing literature and clinician consultation. Subsequently, likelihood functions will be employed to align simulated outputs with observed melanoma data, facilitating parameter updates via Bayesian inference. This approach enables the synthesis of heterogeneous data and yields uncertainty estimates for model outputs, ensuring a rigorous calibration process. Model refinement will involve iterative diagnostic evaluations and sensitivity analyses 70,71 to produce a well-calibrated model that accurately represents melanoma epidemiology”.

Typo’s:

- Page 4, Line 1: “charact eristics” -- > “characteristics”

- Page 4: bracket not closed “(2020/ETH03109; data accessed from 01/01/2022.”

- Page 4, correct punctuation? “melanomas; Fig 1)”

All typos have been corrected.

Reviewer 3

This protocol paper outlines a microsimulation model for assessing the outcomes, costs, and cost-effectiveness of risk-stratified melanoma screening. Melanoma development and detection vary based on histological categories, as well as individual risk factors such as age, sex, and birth cohort. The paper underscores the global relevance and importance of evaluating melanoma screening programs and describes the model specifically within the Australian context.

The revisions to this paper have thoroughly addressed and incorporated the comments from Reviewers #1 and #2. The paper provides detailed information on the microsimulation model and the approach to evaluating the cost-effectiveness of melanoma screening.

We thank the reviewer for the helpful feedback and suggestions.

Two clarifications are worth considering for this manuscript:

Page 10: The probability of melanoma diagnosis is noted to depend on the location of the tumour (in addition to stage and histology). Further information on how tumour location will be incorporated into the model would be beneficial.

To incorporate tumour location into the model, we will assign location using appropriate probability distributions derived from relevant epidemiological data, ensuring accurate representation of location-specific melanoma diagnosis probabilities alongside stage and histology.

We updated the text (page 10) as follows:

“The probability of melanoma being diagnosed at a screening event depends upon several factors including the stage, histology, and location of the undiagnosed tumour (location is assigned using appropriate probability distributions derived from relevant epidemiological data), as well as the sensitivity and specificity of any diagnostic procedures involved.”

Page 12: Keratinocyte cancers and benign lesions are not included in the initial iteration of the model, but their diagnoses will be considered in the melanoma screening cost-effectiveness evaluation. Additional explanation would be helpful to clarify how these non-modelled components will be incorporated into the evaluation. It may be worth moving this paragraph earlier in the paper, as this information relates to the incidence and detection of skin cancers more generally.

In the melanoma screening cost-effectiveness evaluations, keratinocyte cancers and benign lesions, although not explicitly included in Policy1-Melanoma, will be accounted for in the cost estimates. Specifically, for each simulated skin check, we will incorporate the associated costs of keratinocyte cancers and benign lesions based on their expected prevalence and treatment expenses. These costs will be integrated into the overall cost-effectiveness analysis to ensure a comprehensive evaluation.

We updated the text (page 13) as follows:

“Keratinocyte cancers and benign lesions will not be explicitly simulated in the initial iteration of the model. However, when evaluating the cost-effectiveness of risk-based melanoma screening programs, we will consider the financial implications of diagnosing these cancers and lesions in individuals participating in the program. Specifically, for each simulated skin check, we will incorporate the associated costs of keratinocyte cancers and benign lesions based on their expected prevalence and treatment expenses.72 Keratinocyte cancers and benign lesions will be incorporated into subsequent versions of the model.”

Attachment Submitted filename: ResponsesToReviewers_2025-05-24.docx

# Peer review: Decision Letter 2

**DOI:** 10.1371/journal.pone.0339177.r005

**Authors:** Wenpeng You

8 Jul 2025

Dear Dr. Cust,

Thank you for submitting your manuscript to PLOS ONE. After careful consideration, we feel that it has merit but does not fully meet PLOS ONE’s publication criteria as it currently stands. Therefore, we invite you to submit a revised version of the manuscript that addresses the points raised during the review process.

Please submit your revised manuscript by Aug 22 2025 11:59PM. If you will need more time than this to complete your revisions, please reply to this message or contact the journal office at plosone@plos.org . When you're ready to submit your revision, log on to https://www.editorialmanager.com/pone/ and select the 'Submissions Needing Revision' folder to locate your manuscript file.

- A rebuttal letter that responds to each point raised by the academic editor and reviewer(s). You should upload this letter as a separate file labeled 'Response to Reviewers'.
- A marked-up copy of your manuscript that highlights changes made to the original version. You should upload this as a separate file labeled 'Revised Manuscript with Track Changes'.
- An unmarked version of your revised paper without tracked changes. You should upload this as a separate file labeled 'Manuscript'.

If applicable, we recommend that you deposit your laboratory protocols in protocols.io to enhance the reproducibility of your results. Protocols.io assigns your protocol its own identifier (DOI) so that it can be cited independently in the future. For instructions see: https://journals.plos.org/plosone/s/submission-guidelines#loc-laboratory-protocols . Additionally, PLOS ONE offers an option for publishing peer-reviewed Lab Protocol articles, which describe protocols hosted on protocols.io. Read more information on sharing protocols at https://plos.org/protocols?utm_medium=editorial-email&utm_source=authorletters&utm_campaign=protocols .

We look forward to receiving your revised manuscript.

Kind regards,

Wenpeng You

Academic Editor

PLOS ONE

Journal Requirements:

Please review your reference list to ensure that it is complete and correct. If you have cited papers that have been retracted, please include the rationale for doing so in the manuscript text, or remove these references and replace them with relevant current references. Any changes to the reference list should be mentioned in the rebuttal letter that accompanies your revised manuscript. If you need to cite a retracted article, indicate the article’s retracted status in the References list and also include a citation and full reference for the retraction notice.

[Note: HTML markup is below. Please do not edit.]

Reviewers' comments:

Reviewer's Responses to Questions

**Comments to the Author**

1. Does the manuscript provide a valid rationale for the proposed study, with clearly identified and justified research questions?

Reviewer #3: Yes

Reviewer #4: Yes

**********

2. Is the protocol technically sound and planned in a manner that will lead to a meaningful outcome and allow testing the stated hypotheses?

Reviewer #3: Yes

Reviewer #4: Yes

**********

3. Is the methodology feasible and described in sufficient detail to allow the work to be replicable?

Reviewer #3: Yes

Reviewer #4: No

**********

4. Have the authors described where all data underlying the findings will be made available when the study is complete?

The PLOS Data policy

Reviewer #3: Yes

Reviewer #4: Yes

**********

5. Is the manuscript presented in an intelligible fashion and written in standard English?

Reviewer #3: Yes

Reviewer #4: Yes

**********

Please use the space provided to explain your answers to the questions above and, if applicable, provide comments about issues authors must address before this protocol can be accepted for publication. You may also include additional comments for the author, including concerns about research or publication ethics.

You may also provide optional suggestions and comments to authors that they might find helpful in planning their study.

Reviewer #3: Thanks for addressing the remaining queries. I look forward to seeing this valuable protocol published.

Reviewer #4: Dear authors,

Thank you for the opportunity to review this nice paper. Please find my comments below, hope these can be helpful to you to improve the quality of your manuscript.

• You are presenting a protocol for the development, calibration, and validation of a microsimulation platform to predict melanoma burden. Can this protocol be used by any country, other than Australia, given they have or can approximate the necessary inputs at the national level? You can respond by giving reference to Table 1, and perhaps better, include that information in Table 1 itself, since its title has “Australia” in it which suggests that the protocol and model assumptions , and particularly the model structure are more specific to Australia. I suppose the melanoma natural disease history models should be more generic, and distinction between countries may appear either in parameter values, or some policy/ scenario feasibilities.. etc, but less for structural elements of the model.

• Your natural disease history model for melanoma: How does it compare with existing natural disease history models for melanoma? Can you provide any references to existing study/ protocol/ modeling papers related to melanoma modeling? I remember some as part of MCED modeling studies, as well as some single cancer screening papers. You can perhaps add mention these references by making reference to your main Figure 1.

• Your natural disease history and disease progression diagram is an important figure, can you increase resolution to make it more readable? And show potential “recovery” on the figure if it doesn’t get it very crowded? Now it feels like people don’t recover, and everybody gets diagnosed, there are also “undiagnosed” ones..

• Keywords: you may add a few mesh-terms instead of the existing ones which are not mesh-terms, e.g. “early detection of cancer”, see for others: https://www.ncbi.nlm.nih.gov/mesh/

• Melanoma incidence: The global incidence is 2%, as we understand. You are stating that “This is particularly true in Australia and New Zealand, where despite incidence rates in young people stabilising, melanoma incidence remains the highest in the world”. Can you state the prevalence of the disease specifically, e.g. is it 4%? And perhaps same here, where you mention the incidence rates: “….which have been credited with reducing melanoma incidence in people under the age of 40 years”

• Some of the text can be stated as a footnote, or moved to Appendix. E.g. this info can be a footnote. “…Tumours are classified using ICD-O-3 for body sites C44.0-C44.9 (and C80.9 melanoma of unknown primary site if not coded C44.9) and morphology codes M872-M879 with behaviour code 2 (in situ) or 3 (invasive).”

• Abbreviations: You have already defined USPSTF, the U.S. Preventive Services Task Force, on page 15, you do not need to spell it out in the text after that, try to stick to a standardized use, and for all abbreviations. It is spelled as U.S. and not US in text.

• You can say “expert opinion “we will estimate this through discussion with clinicians and from cohort studies”.

• Add a “List of Abbreviations” , since you have used many in text.

We need to have more of these modeling papers/ protocols on cancer early detection, particularly for understudied types of cancers. I wish you all best with this study.

**********

what does this mean? ). If published, this will include your full peer review and any attached files.

If you choose “no”, your identity will remain anonymous but your review may still be made public.

**Do you want your identity to be public for this peer review?** For information about this choice, including consent withdrawal, please see our Privacy Policy

Reviewer #3: No

Reviewer #4: No

**********

[NOTE: If reviewer comments were submitted as an attachment file, they will be attached to this email and accessible via the submission site. Please log into your account, locate the manuscript record, and check for the action link "View Attachments". If this link does not appear, there are no attachment files.]

While revising your submission, please upload your figure files to the Preflight Analysis and Conversion Engine (PACE) digital diagnostic tool, https://pacev2.apexcovantage.com/ . PACE helps ensure that figures meet PLOS requirements. To use PACE, you must first register as a user. Registration is free. Then, login and navigate to the UPLOAD tab, where you will find detailed instructions on how to use the tool. If you encounter any issues or have any questions when using PACE, please email PLOS at figures@plos.org

# Author comment: Author response to Decision Letter 3

**DOI:** 10.1371/journal.pone.0339177.r006

8 Aug 2025

Note – there were no comments from Reviewer 1 or Reviewer 2.

Reviewer 3

Thanks for addressing the remaining queries. I look forward to seeing this valuable protocol published.

No changes required.

Reviewer 4

1. You are presenting a protocol for the development, calibration, and validation of a microsimulation platform to predict melanoma burden. Can this protocol be used by any country, other than Australia, given they have or can approximate the necessary inputs at the national level? You can respond by giving reference to Table 1, and perhaps better, include that information in Table 1 itself, since its title has “Australia” in it which suggests that the protocol and model assumptions , and particularly the model structure are more specific to Australia. I suppose the melanoma natural disease history models should be more generic, and distinction between countries may appear either in parameter values, or some policy/ scenario feasibilities.. etc, but less for structural elements of the model.

We agree with the reviewer that melanoma natural history disease models should ideally be generalisable across contexts. However, the transition assumptions in Table 1 of our protocol are informed by Australian data. It is possible that some parameters will vary across countries, given different UV exposure, incidence and mortality rates, public health campaigns and sun-related behaviours. For the protocol to be applied in other countries, these assumptions must be validated using country-specific data. Thus, the protocol is transferable provided that relevant local data are available and the transition assumptions in Table 1, validated for Australia, are confirmed to be applicable.

We added the following to the Discussion section (page 15): "While the proposed protocol is tailored to the Australian context, its structure can be adapted for other countries by validating the transition assumptions in Table 1 and parameterising the model with country-specific data.”

2. Your natural disease history model for melanoma: How does it compare with existing natural disease history models for melanoma? Can you provide any references to existing study/ protocol/ modeling papers related to melanoma modeling? I remember some as part of MCED modeling studies, as well as some single cancer screening papers. You can perhaps add mention these references by making reference to your main Figure 1.

In a recently published systematic review (https://doi.org/10.1186/s12911-025-03074-9), we have looked at published microsimulation models of skin cancer and conducted a narrative synthesis of these models to assess their structures, parametrisation and assumptions. The structure we propose in this protocol paper addresses several limitations identified in our review. We added the following sentence to the Methods and Analysis section (bottom of page 4): “This natural history structure addresses key limitations identified in a recent systematic review, which synthesised the structures, parameterisation, and assumptions of existing models.18”

3. Your natural disease history and disease progression diagram is an important figure, can you increase resolution to make it more readable? And show potential “recovery” on the figure if it doesn’t get it very crowded? Now it feels like people don’t recover, and everybody gets diagnosed, there are also “undiagnosed” ones..

We have now increased the resolution of the image. We appreciate the reviewer’s suggestion to explicitly depict “recovery” and “undiagnosed” cases in the natural history diagram to better reflect melanoma outcomes. In the current diagram (Figure 1), recovery is implicitly captured, as individuals who do not die from melanoma remain in earlier health states. Similarly, undiagnosed cases are incorporated within the histological states, as the model does not assume universal disease diagnosis. We believe these implicit representations, described in Figure 1’s caption, maintain the diagram’s clarity and simplicity while accurately reflecting the disease’s natural history. We considered adding ‘death from other causes’ to the figure, but this would need to be added in multiple places, making the figure very crowded and more difficult to read.

4. Keywords: you may add a few mesh-terms instead of the existing ones which are not mesh-terms, e.g. “early detection of cancer”, see for others: https://www.ncbi.nlm.nih.gov/mesh/

We have now added MeSH terms as Keywords (page 2).

5. Melanoma incidence: The global incidence is 2%, as we understand. You are stating that “This is particularly true in Australia and New Zealand, where despite incidence rates in young people stabilising, melanoma incidence remains the highest in the world”. Can you state the prevalence of the disease specifically, e.g. is it 4%? And perhaps same here, where you mention the incidence rates: “….which have been credited with reducing melanoma incidence in people under the age of 40 years”

We have addressed this comment by adding more details to the text:

Page 3: “This is particularly true in Australia, where melanoma incidence remains the highest in the world3 and represents 11% of new invasive cancer diagnoses.11”

Page 6: “Since 1980, Australia has implemented many public health programs to reduce sun exposure, which have been credited with reducing melanoma incidence in people under the age of 40 years.29,30 Some of the reduction in incidence may also be due to changes in the ancestral composition of the Australian population.31 Nevertheless, despite the risk of being diagnosed with melanoma by the age of 30 in Australia halving since 1982, it remains the most common cancer among young adults aged 20-39 years and represents 16% of new invasive cancer diagnoses for that age-group.11

6. Some of the text can be stated as a footnote, or moved to Appendix. E.g. this info can be a footnote. “…Tumours are classified using ICD-O-3 for body sites C44.0-C44.9 (and C80.9 melanoma of unknown primary site if not coded C44.9) and morphology codes M872-M879 with behaviour code 2 (in situ) or 3 (invasive).”

We have moved this text to the Figure 1 description.

7. Abbreviations: You have already defined USPSTF, the U.S. Preventive Services Task Force, on page 15, you do not need to spell it out in the text after that, try to stick to a standardized use, and for all abbreviations. It is spelled as U.S. and not US in text.

We have removed the abbreviation USPSTF since it only appeared twice, and changed US to U.S.

8. You can say “expert opinion “we will estimate this through discussion with clinicians and from cohort studies”.

We have changed “discussion with clinicians” to “expert opinion”.

9. Add a “List of Abbreviations” , since you have used many in text.

Rather than provide a list of abbreviations, we have removed most abbreviations in the text to make it easier to read.

10. We need to have more of these modeling papers/ protocols on cancer early detection, particularly for understudied types of cancers. I wish you all best with this study.

Thank you!

Attachment Submitted filename: ResponseToReviewers_20250807.docx

# Peer review: Decision Letter 3

**DOI:** 10.1371/journal.pone.0339177.r007

**Authors:** Yury Khudyakov

2 Dec 2025

A protocol for development of a microsimulation model platform to evaluate the potential benefits, harms, and cost-effectiveness of risk-tailored melanoma screening

PONE-D-24-40002R3

Dear Dr. Cust,

We’re pleased to inform you that your manuscript has been judged scientifically suitable for publication and will be formally accepted for publication once it meets all outstanding technical requirements.

Within one week, you’ll receive an e-mail detailing the required amendments. When these have been addressed, you’ll receive a formal acceptance letter and your manuscript will be scheduled for publication.

An invoice will be generated when your article is formally accepted. Please note, if your institution has a publishing partnership with PLOS and your article meets the relevant criteria, all or part of your publication costs will be covered. Please make sure your user information is up-to-date by logging into Editorial Manager at Editorial Manager®  and clicking the ‘Update My Information' link at the top of the page. For questions related to billing, please contact billing support .

If your institution or institutions have a press office, please notify them about your upcoming paper to help maximize its impact. If they’ll be preparing press materials, please inform our press team as soon as possible -- no later than 48 hours after receiving the formal acceptance. Your manuscript will remain under strict press embargo until 2 pm Eastern Time on the date of publication. For more information, please contact onepress@plos.org.

Kind regards,

Yury E Khudyakov, PhD

Academic Editor

PLOS ONE

Additional Editor Comments (optional):

Reviewers' comments:

Reviewer's Responses to Questions

**Comments to the Author**

1. Does the manuscript provide a valid rationale for the proposed study, with clearly identified and justified research questions?

Reviewer #3: Yes

**********

2. Is the protocol technically sound and planned in a manner that will lead to a meaningful outcome and allow testing the stated hypotheses?

Reviewer #3: Yes

**********

3. Is the methodology feasible and described in sufficient detail to allow the work to be replicable?

Reviewer #3: Yes

**********

4. Have the authors described where all data underlying the findings will be made available when the study is complete?

The PLOS Data policy

Reviewer #3: Yes

**********

5. Is the manuscript presented in an intelligible fashion and written in standard English?

Reviewer #3: Yes

**********

Please use the space provided to explain your answers to the questions above and, if applicable, provide comments about issues authors must address before this protocol can be accepted for publication. You may also include additional comments for the author, including concerns about research or publication ethics.

You may also provide optional suggestions and comments to authors that they might find helpful in planning their study.

Reviewer #3: All of the reviewer's comments appear to have been well addressed by the authors. This protocol paper is ready for publication.

**********

what does this mean? ). If published, this will include your full peer review and any attached files.

If you choose “no”, your identity will remain anonymous but your review may still be made public.

**Do you want your identity to be public for this peer review?** For information about this choice, including consent withdrawal, please see our Privacy Policy

Reviewer #3: No

**********

# Editor report: Acceptance letter

**DOI:** 10.1371/journal.pone.0339177.r008

**Authors:** Yury Khudyakov

PONE-D-24-40002R3

PLOS One

Dear Dr. Cust,

I'm pleased to inform you that your manuscript has been deemed suitable for publication in PLOS One. Congratulations! Your manuscript is now being handed over to our production team.

At this stage, our production department will prepare your paper for publication. This includes ensuring the following:

* All references, tables, and figures are properly cited

* All relevant supporting information is included in the manuscript submission,

* There are no issues that prevent the paper from being properly typeset

You will receive further instructions from the production team, including instructions on how to review your proof when it is ready. Please keep in mind that we are working through a large volume of accepted articles, so please give us a few days to review your paper and let you know the next and final steps.

Lastly, if your institution or institutions have a press office, please let them know about your upcoming paper now to help maximize its impact. If they'll be preparing press materials, please inform our press team within the next 48 hours. Your manuscript will remain under strict press embargo until 2 pm Eastern Time on the date of publication. For more information, please contact onepress@plos.org.

You will receive an invoice from PLOS for your publication fee after your manuscript has reached the completed accept phase. If you receive an email requesting payment before acceptance or for any other service, this may be a phishing scheme. Learn how to identify phishing emails and protect your accounts at https://explore.plos.org/phishing.

If we can help with anything else, please email us at customercare@plos.org.

Thank you for submitting your work to PLOS ONE and supporting open access.

Kind regards,

PLOS ONE Editorial Office Staff

on behalf of

Dr. Yury E Khudyakov

Academic Editor

PLOS One
